# Predictive model of transcriptional elongation control identifies trans regulatory factors from chromatin signatures

**DOI:** 10.1093/nar/gkac1272

**Published:** 2023-02-02

**Authors:** Toray S Akcan, Sergey Vilov, Matthias Heinig

**Affiliations:** Institute of Computational Biology, Helmholtz Zentrum München Deutsches Forschungszentrum für Gesundheit und Umwelt (GmbH), Ingolstädter Landstraße 1, 85764 Neuherberg, Germany; Department of Computer Science, TUM School of Computation, Information and Technology, Technical University Munich, Munich, Germany; Institute of Computational Biology, Helmholtz Zentrum München Deutsches Forschungszentrum für Gesundheit und Umwelt (GmbH), Ingolstädter Landstraße 1, 85764 Neuherberg, Germany; Institute of Computational Biology, Helmholtz Zentrum München Deutsches Forschungszentrum für Gesundheit und Umwelt (GmbH), Ingolstädter Landstraße 1, 85764 Neuherberg, Germany; Department of Computer Science, TUM School of Computation, Information and Technology, Technical University Munich, Munich, Germany; DZHK (German Centre for Cardiovascular Research), Munich Heart Association, Partner Site Munich, 10785 Berlin, Germany

## Abstract

Promoter-proximal Polymerase II (Pol II) pausing is a key rate-limiting step for gene expression. DNA and RNA-binding trans-acting factors regulating the extent of pausing have been identified. However, we lack a quantitative model of how interactions of these factors determine pausing, therefore the relative importance of implicated factors is unknown. Moreover, previously unknown regulators might exist. Here we address this gap with a machine learning model that accurately predicts the extent of promoter-proximal Pol II pausing from large-scale genome and transcriptome binding maps and gene annotation and sequence composition features. We demonstrate high accuracy and generalizability of the model by validation on an independent cell line which reveals the model's cell line agnostic character. Model interpretation in light of prior knowledge about molecular functions of regulatory factors confirms the interconnection of pausing with other RNA processing steps. Harnessing underlying feature contributions, we assess the relative importance of each factor, quantify their predictive effects and systematically identify previously unknown regulators of pausing. We additionally identify 16 previously unknown 7SK ncRNA interacting RNA-binding proteins predictive of pausing. Our work provides a framework to further our understanding of the regulation of the critical early steps in transcriptional elongation.

## Graphical Abstract

**Graphical Abstract:**
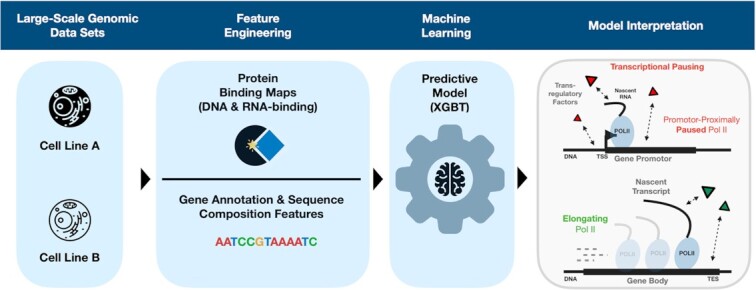
Predictive modeling of transcriptional elongation control based on large-scale protein binding maps, gene annotation and sequence composition features in multiple cell-lines followed by model interpretation reveals trans regulatory factors.

## INTRODUCTION

Transcription of genes is an essential mechanism to maintain cell homeostasis and enable adaptation to changing internal and external stimuli (1,2). It is tightly regulated by chromatin state and transcription factors (TFs) functioning in a highly coordinated fashion (3). The transcriptional cycle starts with the recruitment of the RNA polymerase into the pre-initiation complex (PIC) (4,5). During transcription initiation, a short fragment of nascent RNA is synthesized. The polymerase is then paused at the promoter before entering into productive elongation upon further regulatory signals or terminating prematurely (6). This promoter-proximal pausing is a key rate-limiting step for gene expression as it decides whether a full-length transcript will be made or not (7,8). At equilibrium, paused RNA polymerase accumulates at the promoter since the transcriptional initiation rate is faster than the rate of productive elongation or premature termination (9,10). In vivo, this accumulation can be observed in assays that monitor nascent transcription, such as global run-on sequencing (GRO-seq) (11). Based on this data, the equilibrium between transcription initiation and productive elongation, which is decisive for the regulation of gene expression, can be quantified by the pausing index (PI), also known as the traveling ratio (TR) (12–14). It is defined as the ratio of GRO-seq reads in a window around the promoter compared to the rest of the gene body.

Promoter proximal pausing is the default state after transcription initiation (10,15–17). In addition, the duration of pausing is regulated by the interplay of specific factors that either promote pausing or elongation (16). Pause promoting factors include the DSIF complex consisting of SUPT5H and SUPT4H1, the negative elongation factor NELF, the 7SK complex, consisting of the most highly expressed non-coding RNA *7SK* and proteins such as LARP7, and also specific features of the DNA/RNA sequence (7,18–23). The most important elongation-promoting factor is the positive transcription elongation factor B (P-TEFb), which consists of CDK9, CCNK, CCNT1 and CCNT2 (24,25). Biochemical blocking of P-TEFb showed that its activity is critically important for pause release (26–30). Positive and negative regulators are tightly interlinked. P-TEFb is bound by the inactivating 7SK complex and can be released into its active form by BRD4 (31). Once active it phosphorylates regulators of elongation, such as DSIF, as well as other regulators of chromatin state and RNA processing (32). In addition to these direct regulators, pausing is also indirectly regulated by factors that determine transcriptional initiation and transcript processing (33,34). For example, SRSF2 regulates splicing and has been demonstrated to also determine the duration of pausing (35,36).

Recruitment of P-TEFb to specific promoters through interactions with individual TFs (e.g. NFKB), Mediator, coactivators, and RNA-binding proteins (e.g. DDX2, SRSF2) has been described (35,37–39). Large-scale binding maps of hundreds of RNA binding proteins (RBPs) have recently become available from the ENCODE project (40). Together with the DNA binding maps and GRO-seq, these data allow us to systematically address several key questions about the regulation of pausing at specific promoters. First, which sequence or protein factors determine the recruitment of regulators to a specific promoter? Second, how do signals from positive and negative regulators translate into the extent of pausing quantitatively?

Here, we address these questions by training machine learning models that predict the extent of promoter-proximal pausing quantified by the pausing index from large-scale genome and transcriptome binding maps as well as gene annotation and sequence composition features. We demonstrate high accuracy and generalizability of the model by validation on an independent cell line and we show that the model can accurately predict differential pausing between cell lines indicating that the model captured general cell line independent rules of pausing regulation. Model interpretation allows for assessing the relative importance of each factor, quantifying their effects and predictive values, and systematically identifying previously unknown regulators of pausing. Grouping of factor contributions by molecular functions confirmed the strong interconnection of pausing and co-transcriptional splicing and other steps of gene expression. We additionally identified 16 previously unknown *7SK* interacting RBPs predictive of pausing. These novel pause regulators allow for a systematic and targeted investigation of the regulation of pausing at specific promoters in more detail. Moreover, they provide entry points for experimental manipulation (e.g. with knockdown experiments) to assess their downstream effects on pausing and gene expression in general.

## MATERIALS AND METHODS

### Transcript annotations (GENCODE)

To engineer gene-centric features of protein binding events and gene annotation and sequence composition features as predictors in our machine learning models we obtained transcript annotations for protein-coding genes and non-coding RNAs from the GENCODE (41) database for the hg19 (GrCH37) genome build. We obtained 81 745 annotated protein-coding transcripts for 20 167 genes. Of these transcripts, 30 186 (18 889 genes) were supported by RefSeq (42) annotations and selected as high-confidence transcripts for the analysis. From the annotations, we obtained 5-prime, intronic, coding exonic and 3-prime genomic regions for each transcript which served to capture interpretable binding sites when integrating CHIP-seq and eCLIP-seq data sets (see CHIP-seq data integration & eCLIPseq data integration). HUGO gene nomenclatures (HGNC) (43) from GENCODE were used to further annotate the transcripts with their respective gene symbols.

A set of non-coding transcripts was obtained through appropriate filtering of the GENCODE transcript annotation set for transcripts that were annotated as one of *miscRNA*, *miRNA*, *snoRNA*, *snRNA* and *lincRNA* which represent miscellaneous, micro, small nucleolar, small nuclear and long intervening RNA biotypes, respectively. These non-coding transcripts were used to engineer features for the machine learning task as well as other downstream analyses, especially in the context of the *7SK* non-coding RNA (see Identification of *7SK* Interacting Proteins). Analogous to the protein-coding transcripts, the genomic regions (5-prime, intronic, exonic, and 3-prime) of non-coding transcripts were used to create binding site features based on CHIP-seq and eCLIP-seq data sets.

### Transcript quantifications (RNA-seq)

To ensure that only expressed transcripts are considered we obtained pre-processed transcript quantifications from total RNA-seq experiments from the ENCODE (44,45) project for the K562 and HepG2 cell lines for the hg19 (GrCH37) genome build. Each experiment had two biological replicates. The obtained transcript expressions were required to have a valid ENSEMBLE (46) ID, to be annotated in the aforementioned GENCODE and RefSeq transcript annotation set, to be expressed (fragments per kilobase million (FPKM) > 0) in both of the replicates. The FPKMs were log_10_-transformed for downstream analyses. After these filtering steps, we considered 16 403 (K562) and 16 670 (HepG2) of the 30 186 protein-coding transcripts and 2655 and 1950 non-coding transcripts for the K562 and HepG2 cell lines, respectively. The transcript quantifications data sets (tsv-files) were taken from ENCODE experiments ENCSR885DVH (K562) and ENCSR181ZGR (HepG2), with accession numbers of replicated experiments ENCFF424CXV and ENCFF073NHK for the K562 cell line and accession numbers ENCFF205WUQ and ENCFF915JUZ for the HepG2 cell line, respectively.

Transcript quantification for the Hela cell line were taken from GSM2400170 and were processed in analogy to the RNA-seq data sets of the K562 and HepG2 cell lines. We thereby obtained the expression profiles of *n* = 17 934 protein-coding and *n* = 3331 non-coding transcripts in the Hela cell line.

### Transcription start site annotations (CAGE)

To increase the confidence in the expressed transcripts, we further integrated Cap-analysis Gene Expression Data (CAGE) (47) transcription start sites (TSS) for the K562 and HepG2 cell lines. CAGE read counts of the most correlated replicates were aggregated per cell fraction per cell line. Reads were normalized to transcripts per million reads (TPMs). Resulting TSS were then parametrically clustered (48) into CAGE transcription start site clusters (CTSS cluster) with a TPM threshold of 0.1. Singletons with TPM <0.1 were excluded. Only transcripts whose transcription start site (TSS) was also the dominant CAGE transcription start site (CTSS) in a cell-type specific CTSS cluster were retained. We thereby were left with 16 194 and 16 412 protein-coding transcripts in the K562 and HepG2 cell lines, respectively.

### Quantifying promoter-proximal pol II pausing (GRO-seq)

We integrated Global-Run-On-sequencing (GRO-seq) (49) data to quantify transcriptional pausing at protein-coding genes with the commonly used pausing index (PI) also known as the traveling ratio (12,27). The PIs served as targets to be predicted in a machine learning task. GRO-seq captures the nascent fragments that build up during the transcriptional cycle and thereby allows us to assess Pol II productivity based on the nascent RNA fragment output. As it is commonly done in the field, we have defined the PI as the log_2_ ratio of GRO-seq read counts (number of 30 bp reads overlapping at each position) at the transcription start site (TSS) to the GRO-seq read signals in the gene body. To optimize the PI definition we have built pausing indices with varying TSS window sizes and chose the window size maximizing the negative correlation of the PI with the corresponding transcript expressions (Pearson's ρ = −0.68 (K562) and ρ = −0.66 (HepG2); see Supplementary Figure S1 pausing index optimization). This was motivated by the fact that high PIs, representative of transcriptional pausing, should result in low gene expression profiles and vice versa. This led to a sharp TSS window size of 3 bp ranging 1 bp up- and downstream of the TSS while rendering the remaining part of the transcripts as the gene body window. Read lengths of 30 bp (K562, GSM1480325) and at least 25 bp (HepG2, GSM2428726) ensure that the most frequent Pol II pause site and associated components (50) are covered. Each signal (counts of GRO-seq reads within windows) was then normalized by the respective window size. A pseudo count of 1 read was added to each resulting window for the log_2_ transformation when building the ratio. The PI was calculated for each of the 16194 and 16412 expressed protein-coding transcript in a strand-specific manner for the K562 and HepG2 cell line, respectively. Only transcripts that solely contained the DNA base letters (A, T, C, G) along the whole transcript were considered. This further led to the exclusion of 16 and 9 protein-coding transcripts in the K562 and HepG2 cell lines, respectively. This filtering ensures that we exclude reads that might be erroneously mapped such that we capture the full GRO-seq read signals along the remaining transcripts and thereby obtain comparable signal counts. Overlapping protein-coding transcripts were excluded given the fact that corresponding GRO-seq signals can not be uniquely ascribed to a particular transcript and consequently would result in convoluted PI signals. Transcripts that had no GRO-seq signal neither at the TSS nor in the gene body were excluded as well (*n* = 129 in K562; *n* = 196 in HepG2). This has led to the consideration of 8426 and 8260 protein coding transcripts in the K562 and HepG2 cell lines, respectively (see Supplementary Figure S2 for distribution of pausing indices). The corresponding GRO-seq wig-files can be found under GEO accessions GSM1480325 and GSM2428726 for the K562 and HepG2 cell lines, respectively.

The pausing index based on GRO-cap data for the cross-technology evaluation in the K562 cell line was calculated on data obtained from GSM1480322 and processed in analogy to the K562 and HepG2 GRO-seq data sets. The GRO-seq data for the Hela cell with read lengths of 36bp was taken from GSE62046 and also processed in analogy to the K562 and HepG2 data set, providing the pausing index for *n* = 8428 protein-coding transcripts in the Hela cell line.

### DNA binding sites (CHIP-seq)

Chromatin immunoprecipitation sequencing (CHIP-seq) (51) data served to engineer features of gene-centric genomic protein binding events, which were used as input for the machine learning models. These binding sites for DNA binding proteins (DBPs) were obtained from all available CHIP-seq experiments from the ENCODE project for the K562, HepG2 and Hela cell lines for the hg19 (GrCH37) genome build through corresponding peak-called data sets (bed-files). Perturbation experiments were excluded and only optimal (according to irreproducible discovery rate (IDR)) (52) thresholded replicated peaks were considered for downstream analyses to increase the confidence in the obtained binding sites. Experiments with antibodies directly against the factor of interest and newer versioned experiments were prioritized over epitope-tagged and older versioned experiments. We thereby obtained 5041190 (K562), 4138805 (HepG2) and 1010402 (Hela) genomic binding sites for 309 (K562), 211 (HepG2) and 62 (Hela) factors (see Supplementary Tables S1–S3 for CHIP-seq factors per cell line) that served feature engineering purposes (see Feature Engineering). ENCODE CHIP-seq accession numbers for each cell line can be found in Supplementary Tables S4–S6.

### RNA binding sites (eCLIP-seq)

Enhanced crosslinking and immunoprecipitation (eCLIP-seq) (53) data served to build gene-centric transcriptomic protein binding features. Binding sites of all RNA-binding proteins (RBPs) from the ENCODE project for the K562 and HepG2 cell lines were obtained for the hg19 (GrCH37) genome build through corresponding peak-called data sets (bed-files). Perturbation experiments were excluded and only optimal IDR thresholded replicated peaks were considered. Newer versioned experiments were prioritized over older versioned experiments. We thereby obtained 409839 (K562) and 435015 (HepG2) transcriptomic binding sites for 120 (K562) and 103 (HepG2) factors (see Supplementary Tables S7 and S8 of eCLIP-seq factors per cell line) for feature engineering (see Feature Engineering). ENCODE eCLIP-seq accession numbers for each cell line can be found in Supplementary Tables S10 & S11.

Transcriptomic binding sites (*n* = 3 035 169) in the Hela cell line were taken from the POSTAR (54) data base for all available factors (*n* = 30; see Supplementary Table S9) and lifted to the hg19 genome build.

### Identification of *7SK* interacting proteins

We filtered the GENCODE transcript annotation data set for all *7SK* annotated transcripts to enable the identification of known and novel *7SK* binding proteins via observed CLIP-seq signals (eCLIP-seq or POSTAR-derived binding sites) on corresponding transcripts and assess their predictive value in the context of transcriptional pausing. In particular, *7SK* transcripts which were labeled as pseudo versions were included if they were expressed at least at the median expression level of all expressed non-coding transcripts. Their inclusion was motivated by the idea that factors that also bind these pseudo *7SK* transcripts may compete (55) for respective binding sites with factors that bind the non-pseudo version. The set of *7SK* binding factors was defined for each cell line as all factors with at least one CLIP binding site on any of the *7SK* transcripts (see Supplementary Tables S12 - S14).

### Feature engineering

For the machine learning task of predicting the gene-wise pausing index of protein-coding genes we engineered features of DNA- and RNA binding events at protein-coding and the closest proximal non-coding transcripts upstream and downstream of the TSS of each protein-coding transcript. In addition DNA sequence and annotation features of protein-coding transcripts served as predictors for the models. The following features were created:

- transcript length (tx.len)
- strand specification (tx.strand)
- chromosome specification (tx.chr.loc)
- location on the linear genome (tx.loc)
- number of annotated exons (tx.ex.num)
- average exon width (tx.ex.width)
- exon density (tx.ex.ratio; ratio of the length of the transcript including introns to the number of exons)
- fraction of exonic sequence (tx.ex.seq; ratio of the length of all exonic sequences to the transcript length)
- GC content of the whole transcript including introns (tx.gc.seq)
- Width of CAGE transcription start site cluster (CTSS) (tx.tss.width)
- AT content of CTSS (tx.tss.at.cont)
- distance to most proximal CpG island (cpg.island.dist) along with information about the CpG island length (cpg.island.length), and features of the sequence: number of CpGs (cpg.island.count), percentage C or G (cpg.island.percent.cg), percentage of CpG (cpg.island.percent.cpg), and ratio of observed to expected CpG (cpg.island.percent.exp.v.obs))
- binary encoding whether the transcript is a housekeeping gene (housekeeping)
- binary encoding of RBP binding events separately for 5′/3′-UTR, introns and coding exons
- binary encoding of DBP binding events separately for 5′/3′-UTR, introns and coding exons excluding Pol II bindings as these are expected to be naturally correlated with the prediction target
- binary encoding of RBP/DBP binding events separately for 5′/3′-UTR, introns and coding exons of the two most TSS proximal non-coding RNAs excluding polymerase II bindings as these are expected to be naturally correlated with the prediction target

Binary encodings start with either ‘chip’ or ‘clip’, followed by the protein and the genomic or transcriptomic region of the proteins binding events on DNA (‘chip’) or RNA (‘clip’). For instance, ‘chip.RBFOX2.5prime’ denotes a binding event of RBFOX2 on the 5′ end of genomic regions of transcripts. Analogously, ‘clip.RBFOX2.5prime.Proxmial.ncRNA.2’ denotes a binding event of RBFOX2 on the 5′ end of transcriptomic regions of the second most TSS-proximal ncRNA of transcripts. CpG islands have previously been implicated in pausing (56), therefore we included CpG island annotations from the UCSC golden path for the hg19 genome build (cpgIslandExt.txt.gz), to engineer CpG island-centric model features. Annotations of housekeeping genes were taken from (57). The number of proximal ncRNAs was fixed to two since in combination with CHIP-seq and eCLIP-seq signals on these proximal ncRNAs the feature space would otherwise overgrow the number of genes (and therefore data points in the regression task) which would result in overfitting of the models. Numeric features not in the range [0:1] were rescaled to that range to achieve faster and more accurate model convergences. DNA- and RNA-binding signals went into the model as binary features (binding (1) or non-binding (0)) (see Supplementary Tables S15 - S17 for the number of binding events per factor on individual genomic or transcriptomic regions for each cell line). The distribution of annotation-based features for the K562 and HepG2 cell lines can be found in Supplementary Figures S3 and S4, respectively. These feature vectors served as a scaffold to build various data matrices for a machine learning regression task based on different feature sub-spaces defined by prior domain knowledge as discussed in the next section.

### Feature subsets based on prior knowledge

We stratified the feature space into functionally related sets of proteins in order to characterize the relevance and quantify the importance of pre-, co- or post-transcriptional events in the context of transcriptional pausing. These subsets of binding features of DNA- and RNA-binding factors implicated in specific biological processes were constructed by integrating Gene Ontology (GO) (58,59) annotations. Functional sets of factors (Chromatin, Initiation, Elongation, Termination, Splicing) were generated based on whether a specific factor was annotated to a biological process (BP) ontology term of any of the following sets: **Chromatin** (chromosome organization, GO:0051276; chromatin organization, GO:0006325; chromatin remodeling, GO:0006338), **Initiation** (RNA polymerase II preinitiation complex assembly, GO:0051123; transcription initiation from RNA polymerase II promoter, GO:0006367), **Elongation** (transcription elongation from RNA polymerase II promoter, GO:0006368), **Termination** (termination of RNA polymerase II transcription, GO:0006369), **Splicing** (mRNA splicing via spliceosome GO:0045292; regulation of alternative mRNA splicing via spliceosome, GO:0000381) and **Processing** (mRNA export from the nucleus, GO:0006406; mRNA 3′-end processing, GO:0031124). The set of Elongation factors was further extended by pause regulatory factors from the literature (16,60,61) if not already included in the GO-derived factor set **Elongation**. These were super elongation complex (SEC) factors CCNT1, CCNT2, ELL, ELL2, ELL3, AFF1, AFF4, MLLT1, MLLT3, established pausing factors NELFA, NELFB, NELFCD, NELFE, SUPT4H1, SUPT5H, SUPT6H, SUPT16H, BRD4, MYC, TAF1, TBP, PAF1, and CDK9 (P-TEFB), as well as 7SK ncRNA pause mediator complex binding factors LARP7, HEXIM1, HEXIM2 and MEPCE (see also Supplementary Table S13). However, we could only consider a subset (n = 19) of all established pausing factors, which were assayed in the CHIP-seq and eCLIP-seq experiments. The **Elongation** factor set thus contained POLR2A, POLR2B, POLR2G, POLR2H, MLLT1, SUPT5H, GTF2F1, BRD4, WDR43, NCBP2, HNRNPU, LARP7, MYC, TAF1, TBP, AFF1, EZH2, PAF1 and SSRP1. However, polymerase associated factors (POLR2A, POLR2B, POLR2G, POLR2H) were excluded since these are expected to correlate with the pausing signal. A set of *7SK* binding proteins derived from binding sites observed in the eCLIP-seq data was generated to quantitatively assess the importance of unknown or less well-established *7SK*-associated factors (see *7SK* non-coding RNA or Supplementary Tables S12-S14 of *7SK* binding factors per cell line). A set representative of general pausing associated factors was generated by forming the union of the Elongation and *7SK* associated factor set (**Elongation + 7SK**). For a list of factors in each resulting functional factor set per cell line see Supplementary Tables S19 & S20.

Each resulting factor set was further stratified into sequence-specific and non-sequence-specific binders. The Molecular Signatures Database (MSigDB) (62,63), a collection of annotated gene sets, the Catalog of Inferred Sequence Binding Preferences (CIS-BP) (64), a library of transcription factors and their binding motifs, and the Homo sapiens comprehensive model collection (HOCOMOCO) (65), a collection of transcription factor binding models for human and mouse via large-scale ChIP-seq analysis based on binding motifs, were queried to identify sequence specific factors (see Supplementary Tables S21 & S22).

The feature vector space of binding events was then accordingly grouped by these factor sets (see Supplementary Table S23 of factor presence in feature subspaces) to form different feature matrices, always accompanied by DNA sequence and annotation features of protein-coding genes. These feature matrices based on prior domain knowledge, *7SK* ncRNA associations, and sequence-specificity served to build an array of predictive models based on features with a defined biological function. For a baseline comparison of model performances, we have further built 100 random models which randomize over the number of factors, the factors themselves, and their binding patterns. The binding patterns were randomized according to the observed binding proportions.

### Model training

Models of transcriptional pausing were obtained by training Extreme Gradient Boosting Tree (XGB) regressors to predict the pausing index with each of the feature subsets (see previous section). Models were trained in each cell line and validated with (i) a 5-fold cross-validation and the application of the model on a 50% holdout test data set from the same cell line taken at random prior to training (individual models) and with (ii) data from an independent cell line with features that are common to both cell lines (synchronized models). This provided us with an unbiased estimate of the model performances as trained models have neither seen the gene's target distribution nor the specific feature distributions of the other cell line. Although the first validation approach is not based on data from an independent cell line as is the case with the synchronized models, it still provides an unbiased model performance estimate as trained models have also not seen any of the data points from the 50% hold out test data set taken prior to training (cross-validation).

Regression with squared loss was chosen for the learning objective. The coefficient of determination (*R*-squared, *R*^2^) was used as the evaluation metric to compare and evaluate trained models. See Supplementary Table S24 for hyperparameter specification and the Zenodo repository for R-Data structures with all model matrices (model.matrices.RDS).

### Feature scoring

Shapley additive explanations (SHAP) (66,67) were used as a scoring metric for feature contributions. SHAP is a game theoretic approach to explaining the output of any machine learning model. In contrast to the well-known variable importance metric, it is able to show the positive or negative relationship for each feature with the target. As opposed to most feature importance metrics that average over all genes, each gene receives its own set of SHAP values, greatly enhancing the prediction transparency. SHAP values are additive and allow us to aggregate over contributions of subsets of features which enabled us to capture contributions of binding features per protein and subsequently group these proteins into sets of positive and negative regulatory factors. For instance, we obtain contribution scores for a transcription factor binding on the 5′UTR, exons, introns, and 3′UTR on the genome and transcriptome as identified by CHIP-seq and eCLIP-seq, respectively. We derived total factor contributions by aggregating the SHAP scores per factor over each gene region which enabled us to identify specific pause regulatory factors by selecting factors with high effect sizes.

## RESULTS

### Predictive models of transcriptional pausing

**Figure 1.**
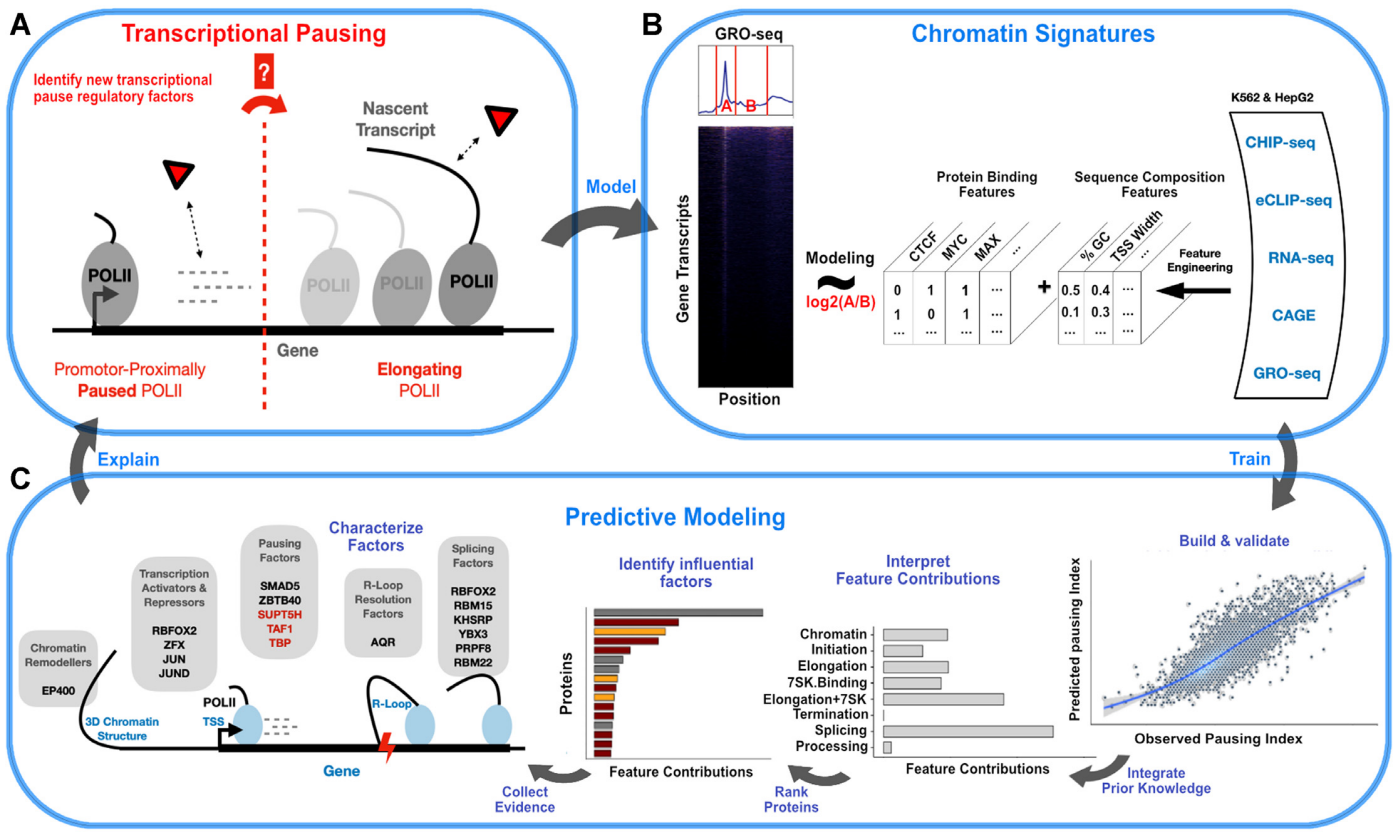
(**A**) Central question as to which specific factors are implicated in the transitioning of promoter-proximally paused polymerase II into its elongating phase of nascent RNA synthesis. (**B**) Integration of large-scale genomic data sets to build the chromatin context of transcriptional pausing (A) with protein binding events and gene annotation and sequence composition features for the prediction task of promoter-proximal pausing of the polymerase II. Pausing is quantified by relating GRO-seq read densities at the TSS to GRO-seq read densities in the gene body. (**C**) Machine learning approach to predict promoter-proximal Pol II pausing with chromatin signatures (B), followed by the integration of prior knowledge and selection of factors as regulators of promoter-proximal Pol II pausing.

The transitioning of promoter-proximally paused Pol II (Figure 1A, promoter-proximally paused Pol II) into its elongating phase of nascent RNA synthesis (Figure 1A, elongating Pol II) is regulated by trans-acting protein co-factors as well as cis-regulatory DNA and RNA sequence features (16,18) which we refer to as chromatin signatures.

For the identification of such specific regulatory chromatin signatures, we used large-scale genomic and transcriptomic protein binding maps from ENCODE and compiled gene annotation and sequence composition features. We then followed a systematic machine learning approach to predict the degree of transcriptional pausing at protein-coding genes (Figure 1B) through the integration of these chromatin signatures in a regression model with Extreme Gradient Boosting trees (XGB) with the potential to reveal explanatory factors (Figure 1C).

**Figure 2.**
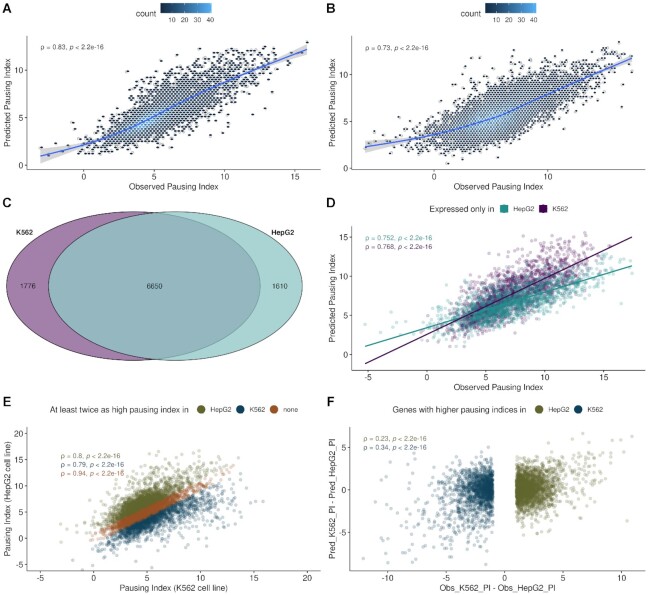
(**A**) Observed versus predicted pausing indices (log2 scale) of a 5-fold cross-validated and regularized XGB regression model in the K562 cell line applied to an independent 50% hold-out test dataset from the same cell line taken prior to training. Pearson's correlation coefficient rho (ρ) with the associated p-value is depicted in the upper left. (**B**) Observed vs. predicted pausing indices of a 5-fold cross-validated and regularized XGB regression model in the K562 cell line applied to the independent test dataset from the cross cell line (HepG2). The model was trained with features common to both cell lines. Pearson's correlation coefficient rho (ρ) with the associated p-value is depicted in the upper left. (**C**) Venn diagram of transcripts between cell lines. (**D**) Observed vs. predicted pausing indices of a 5-fold cross-validated and regularized XGB regression model from each cell line applied to data of genes exclusively expressed in the cross cell line. Pearson's correlation coefficient rho (ρ) with the associated *P*-values are depicted in the upper left. (**E**) Observed pausing indices from the K562 versus HepG2 cell line. Transcripts with at least a 2-fold higher pausing index in one but not the other cell line are colored either green (HepG2 specific transcripts) or blue (K562 specific transcripts). Transcripts with similar pausing indices (less than a 2-fold change) in both cell lines, thus not specific to any of the cell lines, are colored in orange. Pearson's correlation coefficients (ρ) for each of the groups with associated p-values are depicted in the upper left. (**F**) Observed pausing index differences between cell lines against differences of predicted pausing indices obtained from models trained in each cell line and applied to data from the cross cell line. Models were trained on features common to both cell lines. Differences are shown for genes which showed a 2-fold change between cell lines as identified in E).

To facilitate the validation in independent cell lines we obtained relevant data sets for two different cell lines (K562 and HepG2). The prediction target was defined as the gene-wise *pausing index* (see Materials and Methods; see Supplementary Figure S2 for pausing index distributions). It quantifies the degree to which a gene is paused (high pausing index) or elongated (low pausing index). As compared to traditional definitions of the PI, our flexible definition seeks to identify the threshold that is best aligned to the expected relation to transcript levels and covers the more clearly distinguishable peak that lies more proximal to the TSS (see Supplementary Figure S2C–E). To construct the feature matrix of predictors as input for our models we systematically integrated genome-wide CHIP-seq (see Materials & Methods) and eCLIP-seq (see Materials and Methods) data from the ENCODE project, providing DNA and RNA binding sites on the genome and transcriptome respectively (see Supplementary Tables S15 and S16). Gene-centric annotation and composition features were mainly engineered based on GENCODE transcript annotations (see Materials and Methods, Supplementary Figures S3 and S4). CAGE transcription start sites were integrated (see Materials & Methods) to define high confidence TSS and further validate the expression of transcripts. We thereby obtained a total of 2503 features of 2485 DNA & RNA binding and 18 gene annotation features in the K562 cell line and 1832 features of 1814 DNA and RNA binding and 18 gene annotation features in the HepG2 cell line. We then trained an Extreme Gradient Boosting Tree regressor (see Materials and Methods and Supplementary Table S25) to predict the pausing index of protein-coding genes (*n* = 8426 in K562) with high accuracy and explain up to 68% of the observed variance (*R*^2^ ∼ 0.68 on 50% hold-out test data set, K562) of the pausing index (Figure 2A).

The model performances can be further evaluated through (i) the application of a model trained on one cell line and applied to the full data of the other cell line (Figure 2B), (ii) the application of a model trained on one cell line and applied to genes that are only expressed in the other cell line (Figure 2D) and (iii) the application of a model trained on one cell line and applied to genes present in both cell lines with significantly different pausing indices representing extreme observation specific to the other cell line (Figure 2F). See Supplementary Figure S5 for model performances of a model trained on the HepG2 cell line and validated on the K562 cell line.

The predictive power and generalizability of the model were supported by the high prediction performance on the independent cross-cell type test data set (Figure 2B, performance on HepG2 data of K562 model) in which it was still able to explain up to 53% of the variance. The decreased model performance with an *R*^2^ of 0.53 as compared to 0.68 (Figure 2A) is likely due to the reduced amount of features that are available in the HepG2 cell line (39% of all features (*n* = 987) of *n* = 2503 features available in the K562 cell line).

A good performance in the cross-cell type prediction task (Figure 2B) can have two reasons: (i) the model captures the signal of ubiquitously expressed genes that are similar between cell types, as might be the case with housekeeping genes, or (ii) it learned general rules that would also allow for predicting cell type-specific pausing indices from cell type-specific chromatin signatures. To distinguish these scenarios we identified the sets of exclusively expressed genes (Figure 2C) and assessed the performances of models trained on one of the cell lines on the genes exclusively expressed in the other cell line (Figure 2D). The K562 model was able to explain up to 57% and the HepG2 model up to 58% of the observed variance in the pausing indices in the HepG2 and K562 cell line respectively.

We further validated that our model can also identify quantitative changes on transcripts which showed differential (fold change ≥ 2) cell type-specific distributions of the pausing indices. For these sets of transcripts (Figure 2E, blue, green) we evaluated the concordance of observed pausing index differences between the cell lines against the differences in predictions of the pausing indices using models trained in one of the cell lines and applied them to data in the other cell line (Figure 2F). Although we can recognize a substantial decrease in model performances with a correlation of 0.24 (Figure 2F, HepG2 specific pausing indices; green) as compared to 0.73 for the prediction on the entire HepG2 cell type data (Figure 2B) or 0.76 on HepG2 cell type-specific genes (Figure 2D), the model not only predicts extreme cases but also captures quantitative differences of pausing indices specific to the cross cell type to a certain extend. This further underlines the ability of the model to generalize to other cell lines and shows that cross cell type predictions are not only driven by ubiquitously expressed genes.

To further increase the confidence in the obtained modeling results we have additionally investigated (i) data on a third cancer cell line (HeLa), (ii) three additional machine learning methods (Ridge Regression (RR), Random Forests (RF), Gradient Boosting Trees (GBDT)) and (iii) a model based on the pausing index calculated on a different run-on-assay (GRO-cap). These served to additionally validate the cross-cell line prediction performances, rule out prediction performance differences potentially resulting from the selection of the architecture of the machine learning model, and rule out technological bias.

A 5-fold cross-validated and regularized XGB regression model in the Hela cell line (*n* = 92 DNA- and RNA-binding factors) achieves an *R*-squared of 0.56 (Pearson's rho = 0.75) when applied to an independent 50% hold-out test dataset from the same cell line taken prior to training (see Supplementary Figure S6A). This performance is lower than the other full model's performances (*R*-squared HepG2: 0.62, K562: 0.68). The difference can be attributed to the fact that the Hela model includes four times fewer factors than the full K562 model (*n* = 92 versus *n* = 404). Models trained on each cell line using only features present in the HeLa data achieve comparable and expectedly lower model performances of *R*-squared between 0.53–0.56 (see Supplementary Figure S6B). Nevertheless, this reduced set of factors (37/295, only 12% of available factors in HepG2 and 47/404, only 11% of available factors in K562) is still predictive of pausing.

To evaluate the impact of the type of model on prediction performance, we have conducted a systematic comparison with three alternative methods based on the full K562 data set (see Supplementary Table S26), namely Ridge regression (RR), Random Forests (RF), and Gradient Boosting Decision Trees (GBDT). As expected, RR analysis performs worst (*R*-squared 0.6 on K562 50% hold-out test data). The tree-based RF and GBDT models perform similarly well, also compared to the XGB model (*R*-squared RF: 0.69, GBDT: 0.71, XGB: 0.68) and greatly outperform linear regression analysis, as these algorithms can take non-linear relationships into account. The fact that all models perform reasonably well underlines the predictive power of underlying features. These results suggest that the tree based models can be used interchangeably.

To assess whether the model learned a technology bias inherent to GRO-seq, we trained analogous models based on GRO-cap data from K562. The GRO-cap model showed even slightly higher performance (*R*-squared = 0.72, Pearson's rho = 0.85; see Supplementary Figure S7A) than the GRO-seq data (*R*-squared = 0.68, rho = 0.83; see Supplementary Figure S7A) on a hold-out data set of GRO-cap pausing indices. To distinguish if both models learned patterns related to pausing or a technology bias, we applied models trained on one technology to predict the pausing index of the genes in the hold-out test set and compared these predictions to the observed pausing index measured with the second technology (cross-technology evaluation). In this comparison, the GRO-seq model can explain 53% of the variance in the GRO-cap measurements and the GRO-cap model can explain 44% of the variance of the GRO-seq measurements respectively. Given the noise introduced by the different technologies (*R*-squared between GRO-seq and GRO-cap: 0.74) and the uncertainty of the model predictions (*R*-squared GRO-seq model: 0.68), we can calculate the expected proportion of variances explained by the product 0.74 · 0.68 = 0.50. Therefore, the observed *R*-squared of 0.53 (GRO-seq) is well in line with our expectation. These results suggest that the models can generalize between technologies and prediction are not dominated by technology biases.

Given the high predictive power of the obtained model not only on intra-cell type holdout test data sets (Figure 2A), the inter-cell type test data set (Figure 2B) as well as its ability to predict pausing indices of cell type-specific genes (Figure 2D) and cell type-specific differential pausing indices (Figure 2F), we concluded that our model captured general rules of pausing regulation independent of the cell type and that the underlying chromatin signatures of the models would have sufficient discriminatory power to explain the observed variance in the pausing index. The successful validation of model performances on data of a third cell line (see Supplementary Figure S6), with alternative model architectures (see Supplementary Table S26) and an alternative Pol II run-on-assay (see Supplementary Figure S7) further increased the confidence in the obtained modeling results. We thus continued with downstream feature interpretation and selection approaches to suggest potential novel regulators of transcriptional pausing. Downstream analyses were performed on data from the K562 cell line due to the increased amount of features available.

### Contribution of individual transcript processing steps to the prediction of pausing

**Figure 3.**
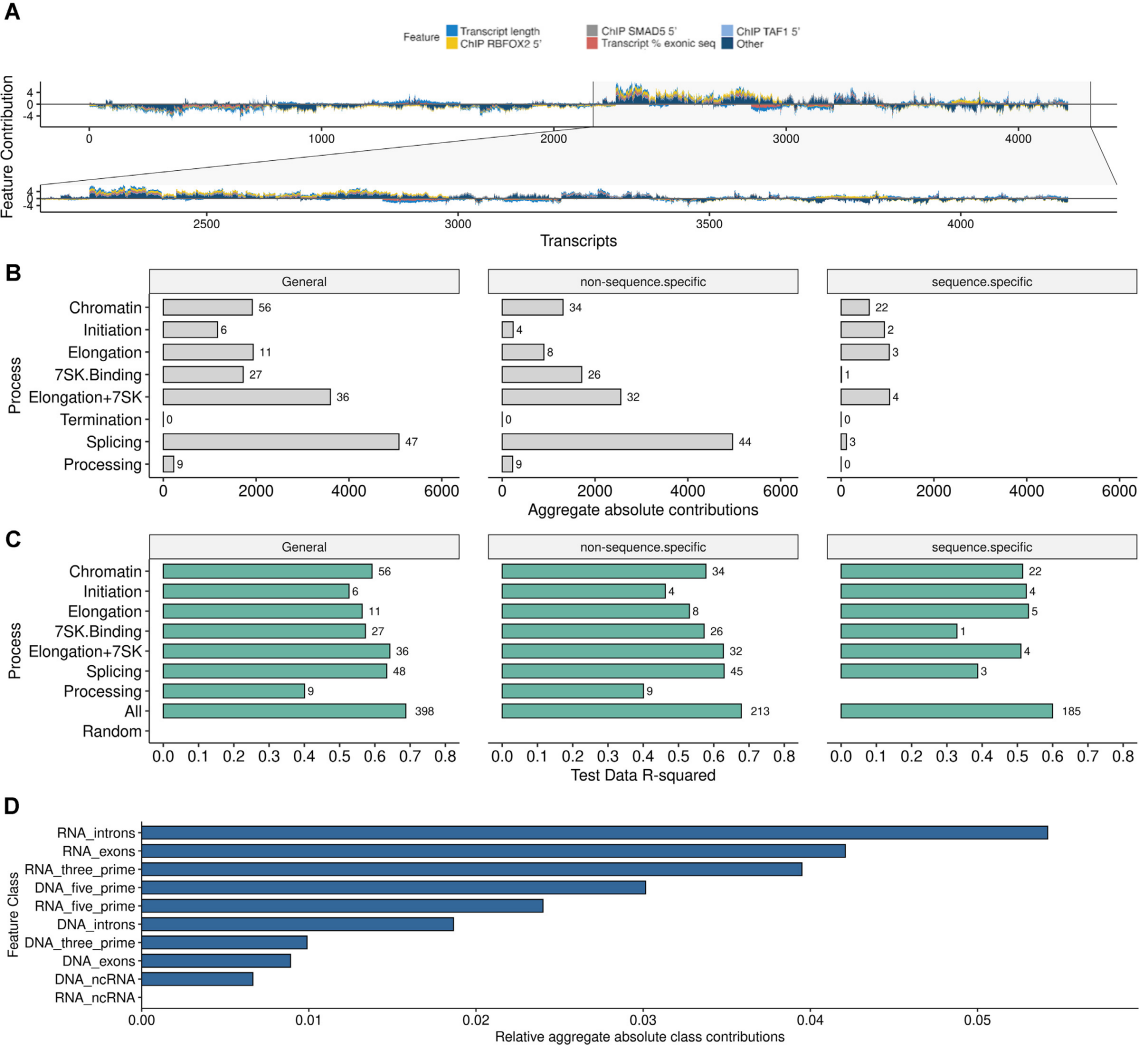
(**A**) Individual feature contributions (SHAP feature contributions, y-axis) on each transcript (x-axis) with a sample zoom-in on a subset of transcripts for better visual investigation. Only the top 5 most influential features are colored and remaining features aggregated in ‘Other’, see legend. Feature ‘ChIP RBFOX2 5′’ refers to the binary indicator variable for a RBFOX2 binding site determined by ChIP-seq being present in the 5′ region of the transcript (see Methods section on feature engineering). The other ChIP-seq data sets are labeled analogously. (**B**) Aggregate absolute contributions of factor classes based on prior knowledge, further divided by sequence and non-sequence specific binding factors. The process ‘*Processing’* refers to mRNA polyadenylation and export from the nucleus. Number of factors are given behind the bars, only factors with non-zero contributions were counted. (**C**) *R*^2^ performances of individual models of factor classes based on prior knowledge on 50% holdout test data set. Number of factors associated with each functional process are given behind the bars, irrespective of their contributions scores, i.e. same factor sets as in (B) which in turn shows only factors with contributions >0. (**D**) Aggregate absolute contributions of factors based on their binding modes.

We next aimed to gain a mechanistic understanding of the underlying predictive contributions. To measure the contributions of model features we have used Shapley Additive Explanations (SHAP) (67,68) as a feature scoring metric (see Materials and Methods) which captures the directional contribution of each model feature specifically for each gene on the target variable. A model feature may increase or decrease the pausing index or exert no effect at all depending on the factors relevance for pausing and their interaction with other features of each gene (Figure 3A). Their combined effects converge in predicted pausing indices which in turn represent the average output whether a gene is paused or not.

Because transcriptional pausing is connected with other steps of gene expression from chromatin organization (69–71), transcription initiation (8,50,72), to splicing (33,73,74) and post-transcriptional transcript processing (34,75,76), we assessed the regulators of these pre-, co- or post-transcriptional events according to their importance in predicting pausing. To that end, we have generated sets of regulators (see Methods and Supplementary Tables S18 - S20) representative of specific RNA processing events (*Chromatin*, *Initiation*, *Elongation*, *Splicing*, *Termination*, *Processing*) based on Gene Ontology (GO) annotations. The *Elongation* factor set was further extended by established pausing factors from the literature. The *7SK* non-coding RNA complex is a key regulator of pausing (35,77–81). To assess the role of RNA binding proteins participating in the 7SK complex for pausing, we additional built a set of factors that bind the *7SK* ncRNA in the eCLIP-seq datasets (see Methods and Supplementary Tables S12 and S13 for *7SK* binding factors per cell line). This set included the well-known *7SK* binder LARP7, the pausing-related regulator AQR previously not associated with the *7SK* as well as the following factors not previously associated with pausing: SSB (LARP3), HNRNPK, DGCR8, PCBP1, ATF, ZNF800, XRCC6, NCBP2, SBDS, YWHAG, GRWD1, ZNF622, SRSF7, TARDBP and BUD13. A set consisting of the union of Elongation and *7SK*-associated factors were generated as well (*Elongation + 7SK*). All sets of regulators were further stratified into known sequence-specific and non-sequence-specific binders (see Supplementary Tables S21 and S22) in order to assess the relevance of sequence-specific binding events. For each factor in the resulting functional set of regulators we aggregated their feature contributions (Figure 3A) per functional process (Figure 3B).

Splicing factors had the highest contributions followed by elongation and *7SK* binding proteins. This strongly supported the intricate connection to co-transcriptional splicing events (36,73,82) and strengthened the role of the newly identified *7SK* binding proteins as transcriptional pause regulatory factors. The *Elongation* factor set of established pausing factors served as a validation of our approach.

We next asked how models would perform if they are trained exclusively on the features defined by each of the previously defined sets of regulators. For a baseline comparison models were also trained on randomized input data (see Materials and Methods). Figure 3C shows the model performances (*R*^2^ values) for each of the feature subspaces of cross-validated models in the K562 cell line on the independent 50% holdout test data sets (see also Supplementary Table S25 for all model results). In general, all models perform reasonably well relative to the number of features they incorporate. As an example, the splicing factor based model (*Splicing*) incorporates only 14% (*n* = 57) of all available factors yet performs almost equally well as the full model (*All*) incorporating all available factors (*n* = 398). Likewise, the *Initiation* model considers only about half the number of factors than the chromatin-associated model (*Chromatin*) yet performs slightly better (*R*^2^ of 0.54 versus 0.53).

As expected, the *7SK* ncRNA-associated factor model (*7SK.Binding*) and the model with previously established pausing factors (*Elongation*) perform very well despite the low number of factors considered in those models. The predictive power of pausing/elongation factors becomes further evident when we consider the model of the union of 7SK and established elongation factors (*Elongation + 7SK*) which outperforms (R^2^ 0.62) each individual factor set alone (*7SK.Binding*: *R*^2^ = 0.55, *Elongation*: *R*^2^ = 0.56) and performs almost equally well as the full model (*R*^2^ = 0.62 versus 0.68). This result highlights the relevance of the novel set of *7SK* binders identified by protein-RNA interactions as putative pause regulators. Taken together, the majority of factor sets show high predictive power relative to the number of factors they incorporate but their performances should not be compared directly to each other due to the variable amount of factors considered in the models. Their predictive value demonstrates the interconnectedness of underlying processes with the transcriptional pausing outcome. It further supported and strengthened the role of the *7SK* ncRNA as a transcriptional pause mediator complex and allowed us to suggest the factors from the set of *7SK* associated factors (*7SK.Binding*) (see Supplementary Tables S12 and S13) as additional *7SK* ncRNA binding proteins to be implicated in the regulation of pausing based on their predictive value.

We next asked whether protein-DNA or protein-RNA binding events contributed to the explanatory power of the models. We found that the individual contributions of RNA binding events are generally higher than those of DNA binding events (Figure 3D). Investigating the contributions of factors by their functional classes within the highest ranked class (RNA introns) (see Supplementary Figure S9 & S10) reveals that splicing factors are enriched for RNA intron binding sites (Fisher's exact test, one-sided (greater), *P* = 0.034, odds ratio 4.45, confidence interval [1.11;Inf] in K562 and *P* = 0.032, odds ratio 7.1 [1.15;Inf] in HepG2). The high contributions of genomic binding events on the 5′ region of transcripts (Figure 3D, DNA_five_prime) are in line with observed 5′ modulated transcriptional pause states (83).

Overall the results for the HepG2 cell line are very similar and support the conclusions (Supplementary Figure S8). Although gene annotation and composition features account for 26% of all feature contributions (see Supplementary Figure S11–S14) they are static in their nature and cannot explain the variation of pausing between cell lines. Therefore, we focus the discussion on individual proteins and their binding events as they are dynamic between cell lines.

### Modulators of transcriptional pausing

**Figure 4.**
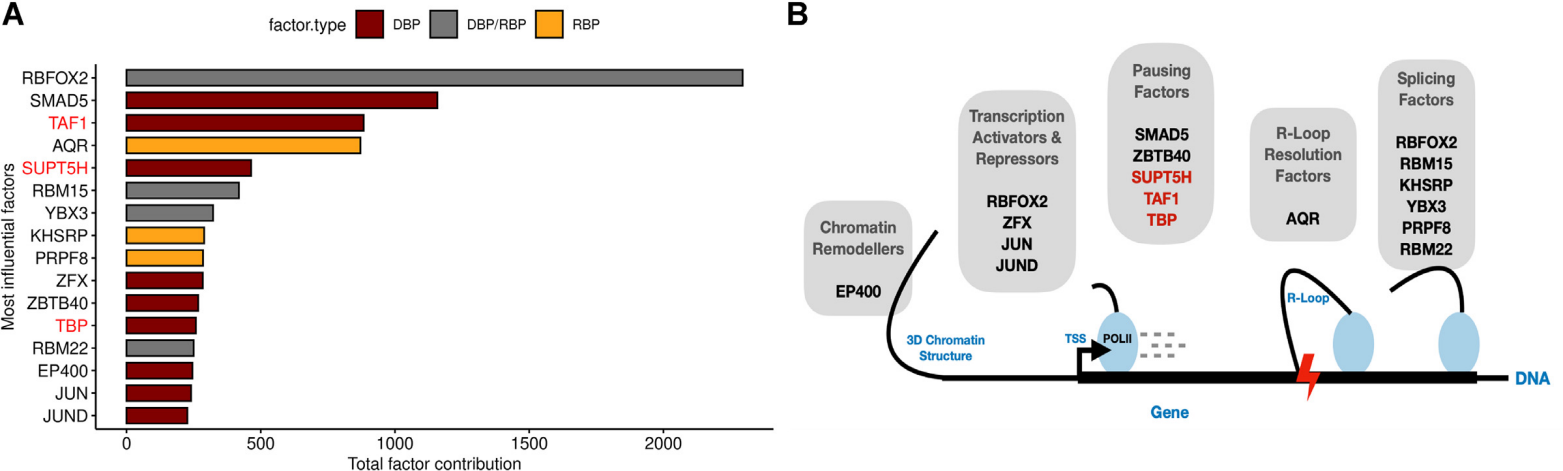
(**A**) Increasingly ordered aggregate factor contributions of factors that make up at least 50% of model contributions. Established pausing/elongation factors are colored red. The bar fill colors identify DNA-binding (DBP; dark red), RNA-binding (RBP; orange), or DNA- and RNA-binding (DBP/RBP; grey) factors. (**B**) A conceptual view on the interconnection and interplay of identified transcriptional pause regulatory proteins with associated transcriptional regulatory processes (Chromatin Remodelling, Transcription Activation/Repression, Transcriptional Pausing, R-Loop resolution and Splicing).

Based on our model, we aimed to identify specific pause regulatory factors. To obtain a ranking of the importance of individual DNA- and RNA-binding factors for predicting Pol II pausing, we aggregated the SHAP contributions (see Supplementary Figures S15 and S16 for individual feature contributions per cell line) into a single contribution score per factor and selected the minimal set of most influential factors (16 out of 398) that makes up 50% of all feature contributions (Figure 4A). Established pausing factors from the literature (Figure 4A, highlighted in red) are ranked among these top influential factors, validating our factor ranking approach. Three factors not primarily related to pausing were ranked higher than the established pausing factors and are potential novel modulators of pausing with at least the effect size of the established factors. However, all other factors have similarly high contributions and can be considered almost equally important.

A minimal model that only operates on the features of these 16 most influential factors (including gene annotation and composition features) which includes only five known pausing or *7SK*-related factors (AQR, BRD4, SUPT5H, TAF1, TBP) achieves an *R*^2^ of 0.65 (on 50% holdout test data set; see Supplementary Figures S17 and S18 performances of minimal models per cell line) and thus performs almost equally well as the full model with all 398 factors and an *R*^2^ of 0.68. Additionally, it outperforms the *Elongation + 7SK* model (Figure 3) which incorporates almost twice as many factors (n = 27) of *7SK*-associated and established elongation factors which, although highly predictive, only achieved an *R*^2^ of 0.61 as compared to an *R*^2^ of 0.65 of the minimal model which indicates that not all pausing related factors were captured in the *Elongation + 7SK* set. The minimal model (*n* = 9) of the HepG2 data consisted of RBFOX2, AQR, TAF1, TBP, RBM15, RBM22 KHSRP, PRPF8 and YBX3, which are all included in the minimal model identified in K562.

To obtain a reference of the predictive power of obtained factors we trained another model solely based on Pol II CHIP-seq binding data (binding patterns of POLR2A, POLR2AphosphoS2, POLR2AphosphoS5, POLR2B, POLR2G and POLR2H) since promoter proximal pausing is tightly related to the phosphorylation state of RNA Pol II which should contain the information necessary to explain the extent of promoter proximal pausing of each transcript and hence be able to predict the extent of pausing defined by the pausing index computed from GRO-seq data. This model (see Supplementary Figure S19) can explain up to 60% of variance in the pausing index as compared to the full K562 model (*n* = 398 factors) which can explain up to 68% or the minimal model (*n* = 16 factors) which can explain up to 65% of variance in the pausing index. The Pol II-only model is missing further subunits (like POLR2C, POLR2E etc.) which is likely the reason why not more of the variance can be explained. However, an additional 8% can be explained by non-polymerase II-associated factors. This is in addition to the 60% of variance explained by polymerase II-associated factors, which underlines non-polymerase II-associated factor's predictive power for transcriptional pausing. This comparison validates our feature engineering, model selection and model training approach, as the model behaves as expected, providing high predictive power. However, for a mechanistic understanding of the regulatory networks in which a variety of factors and co-factors beyond polymerase subunits are interacting to co-modulate different transcriptional processes (e.g. pausing, elongation, splicing) the integration of only polymerase-associated subunits would lead to a circular reasoning and limit the discovery of additional explanatory factors, which necessitates the integration of protein binding data for a broad range of proteins, ideally on the DNA and RNA level.

Lastly, investigating the factor rankings of the previously trained models based on (i) data on of a third cancer cell line (HeLa), (ii) three additional machine learning methods (Ridge Regression (RR), Random Forests (RF), Gradient Boosting Trees (GBDT)) and (iii) the pausing index calculated on a different run-on-assay (GRO-cap), shows that certain factors are consistently ranked high across all validation settings which greatly increases the confidence in the factor's regulatory role in transcriptional pausing. To begin with, a comparison of the top 15 ranking factors (see Supplementary Figure S6 C) from model's trained in each cell line and validated on cross-cell type data with pairwise shared features between the cell lines (see Supplementary Figure S6 B) shows that six factors (TAF1, TBP, UPF1, TIA1, PTBP1 and U2AF2), including the well-established pausing factors (TAF1, TBP) are consistently ranked high across all models. Both included in our minimal factor set (*n* = 16). To continue, a comparison of the tree-based models trained during the evaluation of model architectures (see Supplementary Table S26) shows that 56% (9/16) factors are common among the top 16 ranking factors of trained models. This set consists of RBFOX2, AQR, SMAD5, TAF1, SUPT5H, YBX3, RBM15, KHSRP, and PRPF8. The presence of two well-established pausing factors (TAF1 and SUPT5H) again validates our factor ranking, model building, and selection approach. Moreover, all of these factors are included in our minimal factor model which further increases the robustness of our results as different model architectures converge on a similar ranking of explanatory predictors. Finally, a comparison of the 16 top contributing factors (see Supplementary Figure S7 B) of models trained to predict pausing indices based on different sequencing protocols (GRO-seq versus GRO-cap) (see Supplementary Figure S7 A), with the top ranking factors from the minimal K562 model, shows that 68% (11/16) of these factors are common across both models providing predictive power across both sequencing protocols. This set of factors consists of RBFOX2, SMAD5, TAF1, AQR, SUPT5H RBM15, YBX3, KHSRP, ZFX, TBP and EP400. These factors represent confident regulators of transcriptional pausing as they are selected across different sequencing protocols.

Upon investigation of the identified most influential pausing factors (*n* = 16, K562) defined by our model the interconnection of pausing with other RNA-processing events becomes further apparent. An interesting picture emerges considering the functional background of these factors (Figure 4B).

### Pausing factors

Several pausing factors are well established (TAF1, TBP, SUPT5H) and occupy high ranks in our models. TAF1 and TBP are components of the pre-initiation complex (PIC). Its formation inherently leads to pausing (61). This behavior can be modulated by other pausing factors, especially the protein complexes NELF and DSIF (SUPT5H) increase pausing whereas the P-TEFb complex associates with pause release.

### Chromatin remodelers

The chromatin remodeler EP400 had a large impact on our model. Chromatin state is defined by nucleosome positioning and posttranslational modification of its histones. It is tightly linked to transcription initiation, elongation, and co-transcriptional splicing and can be actively modulated by chromatin remodelers (84–87). EP400 is a histone acetyltransferase and promotes gene activation after PIC assembly through the depositioning of H3.3/H2.AZ into promoters and enhancers (88). It interacts with the well-known pausing factor MYC (27,88,89) and might be linked to transcriptional pausing through this association. In fact, regulation of Pol II pausing at promoter-proximal nucleosomes by chromatin remodelers like for instance CHD1 (90) has already been established.

### Transcriptional repressors and activators

Among the top influential factors we can find activating transcription factors ZFX, JUN, and JUND as well RBFOX2 as a repressive transcription factor. ZFX family members exert a transcription-activating function in multiple types of human tumors and bind downstream from the TSS at the majority of CpG island promoters regulating genes for essential housekeeping functions. ZFX family members have been suggested to act in a similar manner as the MYC family of transcription factors due to their shared pervasive binding at promoter sites as well as similar profound proliferation defects upon knockdown (91,92). Given that MYC plays an important role in transcriptional pause release through the recruitment of P-TEFb (27,93), a similar connection could exist for ZFX. Moreover, a comparison of the binding patterns of ZFX with Pol II and H3K4me3 has shown that ZFX is slightly downstream from the most frequent Pol II pause site and slightly upstream of the downstream peak of the H3K4me3 signal (91,92), further suggesting a role of ZFX in regulating Pol II pausing.

JUN and JUND are subcomponents of the activating protein 1 (AP-1) (94,95) which in turn controls cell proliferation, neoplastic transformation, apoptosis, and the expression of immune mediators. AP-1 is suppressed by the negative elongation factor NELF (96), but so far no regulation of transcriptional pausing by AP-1 has been reported.

RBFOX2 acts both, as a regulator of alternative splicing as discussed later, and transcriptional repressor through the binding to chromatin-associated RNA, especially promoter-proximal nascent RNA, through the recruitment of the polycomb-complex 2 (PRC2) to its site of action (91,97,98). In fact, knockout of *RBFOX2* in cardiomyocytes leads to decreased pausing indices and suggests that RBFOX2 and PRC2 enhance coordinated transcriptional pausing at gene promoters (98).

### Co-transcriptional splicing and mRNA regulatory factors

The presence of several splicing-associated factors (RBFOX2, PRPF8, RBM15, RBM22, KHSRP, YBX3, AQR) further strengthens the intricate connection to co-transcriptional splicing events (33,74,99,100). Co-transcriptional splicing of pre-mRNAs is dependent on the availability of the nascent RNA that forms during the transcriptional cycle which in turn is a function of Pol II pausing. In fact, it has been shown that active spliceosomes are complexed to the Pol II S5P C-terminal domain during elongation and co-transcriptional splicing (101). In particular, it has also been shown that transcription kinetics strongly impact splicing decisions, such that slow Pol II elongation rates allow more time for spliceosome assembly and thereby favor splicing. Moreover, the inhibition of the spliceosomal U2 snRNP function has been shown to enhance Pol II pausing in promoter-proximal regions, impair the recruitment of P-TEFb and thereby reduce Pol II elongation velocity at the beginning of genes (82). These indicated that the release of paused Pol II requires the formation of functional spliceosomes and that positive feedback from the splicing machinery to the transcription machinery exists. In this context, RBFOX2 acts as a well-established regulator of alternative splicing (102–104) with an integral role in transcriptional pausing (98). Likewise, RBM15 (105), RBM22 (106,107), PRPF8 (108), KHSRP (109), and YBX3 (110) as pre-mRNA splicing factors or spliceosome components are likely to have a similar connection to pausing as is the case for RBFOX2 and splicing in general.

AQR is a high-ranking R-loop resolution factor (111). R-loops are RNA/DNA structures in which nascent RNA anneals back to the template DNA (112–115). It has also been suggested that R-loop formation is likely part of the mechanism for Pol II pausing (114) to hold back the elongation of Pol II (116) and the DNA replisome (117). The importance of splicing events for pausing is further strengthened by splice defect-induced R-loop formations as a result of increased RNA-DNA hybrid annealing due to the lack of splicing-dependent nascent RNA processing which would otherwise prevent the formation of such structures through timely splicing events.

### Novel pausing factors

For the factors ZBTB40 and SMAD5 not previously associated with the regulation of pausing we suggest a novel link. ZBTB40 is not well characterized but has been established to be a regulator of osteoblast activity and bone mass (118). SMAD5, together with other SMAD proteins, is a signal transducer and is activated in the cytoplasm and accumulated in the nucleus where it regulates transcription via remodeling of the chromatin architecture through the recruitment of a variety of coactivators and corepressors to the chromatin (91,97), suggesting a role regulating transcriptional pausing outcomes through a series of chromatin remodeling events and recruitment of transcription factors.

## DISCUSSION

The understanding of promoter-proximal Pol II pause regulatory elements is an important step towards disentangling the gene regulatory mechanisms underlying cell homeostasis and plasticity. We improved our understanding by training machine learning models that predict the extent of promoter proximal pausing from large-scale genome and transcriptome binding maps, as well as gene annotation and sequence composition features providing insights into cis- and trans-acting regulatory elements underlying transcriptional pausing. Recent models of transcriptional pausing based on random forests in the Hela cell line (18) focused on NET-seq derived pause sites that are not necessarily promoter proximal. This model solely incorporated DNA sequence features like DNA structures (Z-DNA, repeats etc.), methylation states or transcription factor binding motifs. This is similar to another recent machine-learning approach with a deep-learning architecture called PEPMAN (Feng et al. 2021) to systematically model Pol II pausing events from high-throughput sequencing data based on raw DNA sequence input features. The author's also suggest a strong connection of transcriptional pausing to co-transcriptional splicing events which is very much in line with our results. In contrast to both approaches, our model relies on experimentally determined binding sites of both DNA and RNA binding proteins, which integrate information on the presence of binding sites but also on the cellular context. For example, not all binding motifs are necessarily bound by trans-acting factors in all cell lines.

Our model achieves high predictive accuracy (*R*^2^ ∼ 0.68 with *n* = 389, factors; *R*^2^ ∼ 0.65 with only *n* = 16 factors), indicating that the binding of identified trans-acting protein factors to DNA and RNA explains a large part of the variability of the extent of pausing. The accurate prediction of differential pausing based on cross-cell type specific binding data (*R*^2^ ∼ 0.52) demonstrated that the model learned general rules, which are not cell type specific. This is in line with the observation that the pausing of genes is consistent across a large proportion of cell types (12). Models built from subsets of proteins implicated in all steps of gene expression, including chromatin remodeling, transcription initiation, elongation, splicing, and further downstream transcript processing demonstrated high predictive power. This confirms the intimate cross-talk between these processes (8,16,50,69,70,73,119,120). Of note, factors implicated in splicing have the highest predictive power for pausing. This is in line with many studies that show dual roles for individual proteins such as RBFOX2 (102–104), SRSF2 (33), U2AF65 (82) or MAGOH (82) providing a direct causal link between the two processes. One important goal of our analysis was to identify novel potential pausing regulators. We achieved this using two approaches. First, we identified novel *7SK* binding RBPs and showed that their binding patterns are highly predictive of pausing. Second, we analyzed the feature importance in our model and pinpointed protein factors with higher feature importance than established pausing factors. Many of these factors such as RBOFX2 (102–104), AQR (111), JUN, and JUND (94) have been demonstrated to affect pausing or are implicated in processes that have already been associated with pausing. These factors constitute interesting targets for further experimental validation, as our results already provide some initial mechanistic hypotheses.

We chose to analyze data from the HepG2 and K562 cell lines since they have been extensively characterized in the ENCODE project. The number of DNA and RNA binding maps available is unparalleled and enables the identification of previously unknown regulators of promoter proximal pausing. These data sets come with the limitation that not all previously characterized regulators of pausing are available. The second limitation is that only GRO-seq data and similar variations are available to quantify promoter proximal pausing. Recent multi-omics approaches based on TT-seq (121) and mNET-seq (7,8,122) have been applied to K562 and Raji B cell lines to estimate the kinetic rates of initiation and pause duration more precisely. These approaches provide ground for future studies of transcriptional pausing with greater precision and detail once broadly available across cell lines which would enable elaborate validation procedures. Unfortunately, such data are not available for a second ENCODE cell line such that a cross-validation of the model would not be possible. Taken together, our work provides a framework to further our understanding of the regulation of the critical early steps in transcriptional elongation. We expect further improvements with better kinetic profiling of the polymerase and increasing availability of binding maps or improved prediction of binding sites from sequence.

## DATA AVAILABILITY

The code is available at https://github.com/heiniglab/POLII_pausing. All data and results are also available at 10.5281/zenodo.5236311.

## ACCESSION NUMBERS

GRO-seq data for the K562, HepG2, and Hela cell lines were obtained from studies with GEO accessions ***GSM1480325***, ***GSM2428726*,** and **GSE62046** respectively. GRO-cap data for K562 cell line was taken from ***GSM1480322***. RNA-seq data transcript quantifications data sets (tsv-files) were taken from ENCODE from the experiment **ENCSR885DVH** with accession numbers of replicated experiments **ENCFF424CXV** and **ENCFF073NHK** for the K562 cell line, as well as the experiment **ENCSR181ZGR** with accession numbers of replicated experiments **ENCFF205WUQ**, **ENCFF915JUZ** for the HepG2 cell line. RNA-seq data for the Hela cell line was taken from **GSM2400170**. ENCODE Accession number of CHIP-seq and eCLIP-seq data sets can be found in supplementary tables S4 - S6 and S10 & S11, respectively. Annotations of housekeeping genes were taken from (57) (see Supplementary Table S27; housekeeping.RDS in zenodo repository). CpG island annotations were taken from the UCSC golden path for the hg19 genome build (cpgIslandExt.txt.gz) (see Supplementary Table S27; cpg.islands.RDS in zenodo repository). Gene annotations along with HGNC and RefSeq metadata files were taken from GENCODE (see Supplementary Table S27). CAGE transcription start sites for all cell lines are provided in the zenodo repository as an R-data structure (CTSS.RDS).

## Supplementary Material

gkac1272_Supplemental_Files
