# Supplementary material for: Predictive model of transcriptional elongation control identifies trans regulatory factors from chromatin signatures
Source: Nucleic Acids Res. 2023 Feb 2;51(4):1608–24. doi: 10.1093/nar/gkac1272 (PMC9976927; doi:10.1093/nar/gkac1272)
Supplement: gkac1272_Supplemental_Files [file gkac1272_supplemental_files.zip › Supplementary Data-Transcriptional Pausing.pdf]

Supplementary Materials

Predictive model of transcriptional elongation control identifies trans regulatory factors from chromatin signatures

Toray S. Akcan, Sergey Vilov, Matthias Heinig*

* Corresponding author

Toray S. Akcan
Institute of Computational Biology, Helmholtz Zentrum München, Ingolstädter Landstraße 1, 85764 Neuherberg
Department of Informatics, Technical University Munich
toray.akcan@helmholtz-muenchen.de

Sergey Vilov
Institute of Computational Biology, Helmholtz Zentrum München, Ingolstädter Landstraße 1, 85764 Neuherberg
sergey.vilov@helmholtz-muenchen.de

Matthias Heinig
Institute of Computational Biology, Helmholtz Zentrum München, Ingolstädter Landstraße 1, 85764 Neuherberg
Department of Informatics, Technical University Munich
matthias.heinig@helmholtz-muenchen.de

SUPPLEMENTARY FIGURES

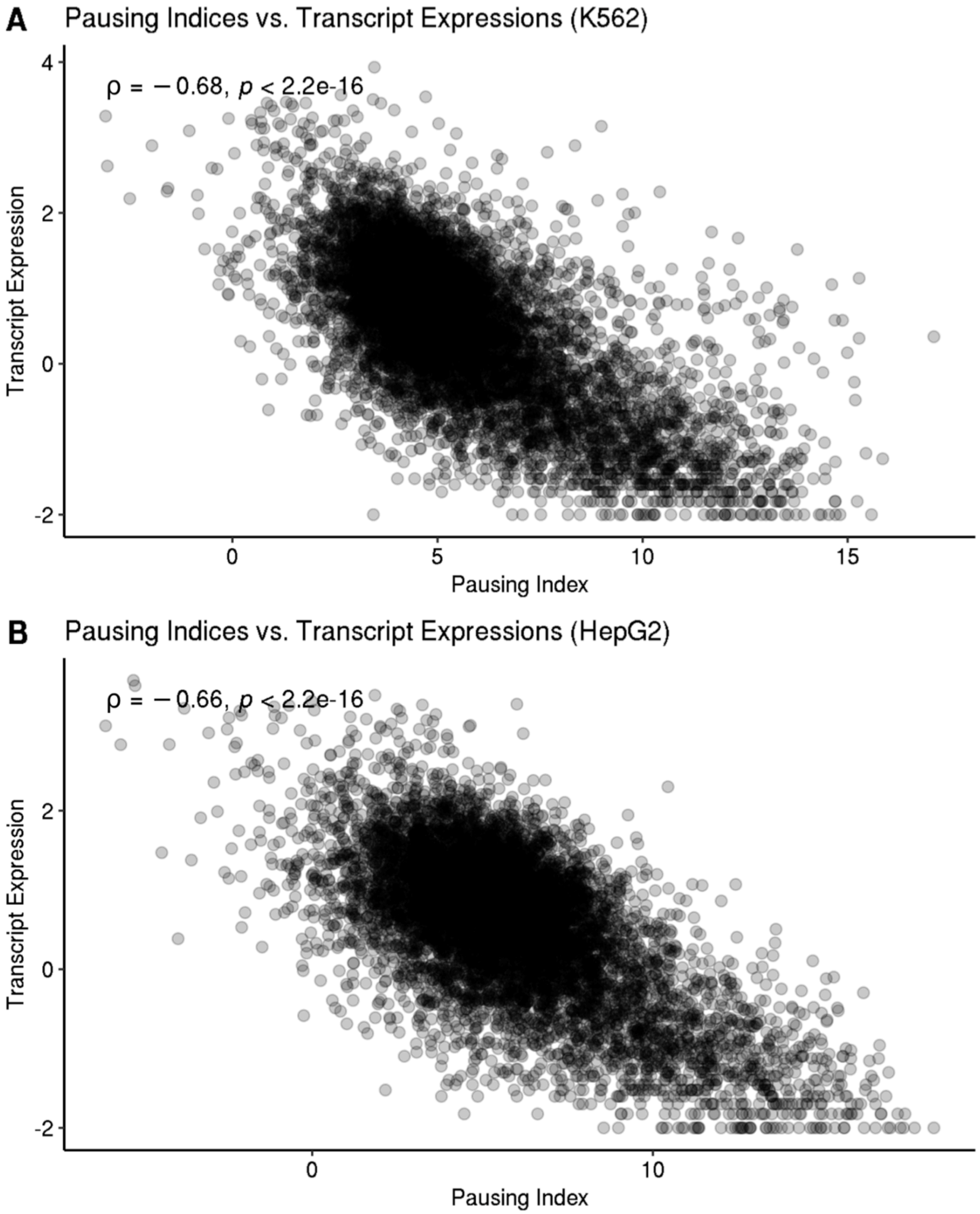

Supplementary Figure 1: Pausing Index Optimization. Inverse correlation of pausing indices (x-axis) with transcript expressions (FPKMs, y-axis) in the K562 (**A**) and HepG2 (**B**) cell line. Pearson's correlation coefficient rho (ρ) with the associated p-value is depicted in the upper left.

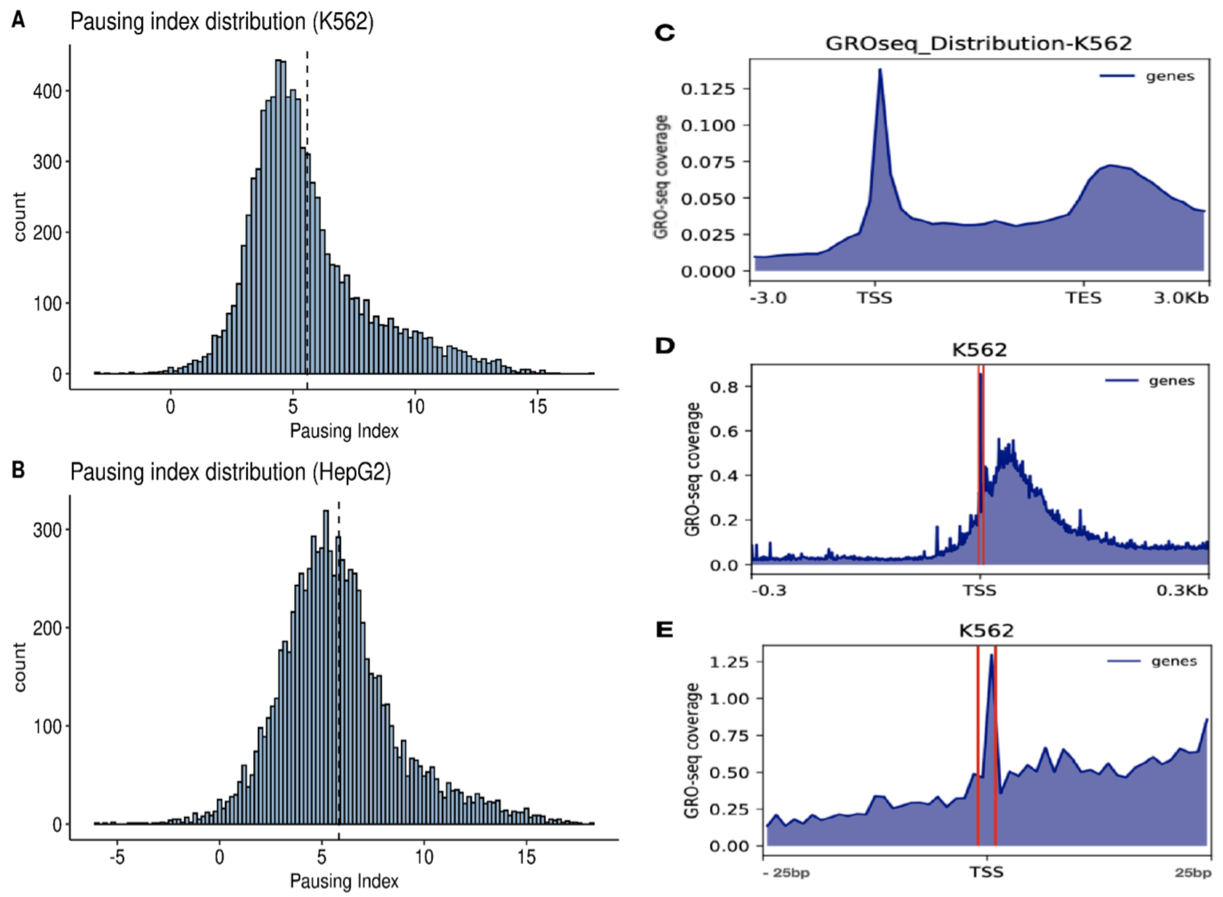

Supplementary Figure 2: The Pausing Index. Histograms of the distribution of pausing indices (PIs) in the K562 (**A**) and HepG2 (**B**) cell line. Dashed lines indicate the mean pausing indices, the x-axes the PIs and the y-axes the PI counts. The pausing index is commonly defined by visual inspection of the global read distribution over meta-transcripts, i.e. the distribution of GRO-seq read signals over the (relative) positions in all protein-coding transcripts (**C**). It clearly shows enrichment of read signals near the TSS as compared to the gene body. This read distribution pattern can be repeatedly recognized in multiple previously published articles concerned with transcriptional pausing, including Daniel S. Day. et al. Genome biology 2016; Core LJ. Cell Rep. 2012. Based on this observation, traditionally, a thresholding approach is applied, defining the pause site region of 50bp upstream and 300bp downstream from the TSS. However, though intuitively it makes sense to contrast this broad peak region at the TSS to the rest of the gene body, it is not known which of the peaks and reads correspond to promoter-proximally paused or for elongation primed polymerases, i.e. it is not known, where to set the threshold for the TSS window which accurately separates promoter from the gene body region. In fact, zooming into the TSS region (+-300bp from TSS) clearly shows that the broad peak region ranging 300bp into the gene body is preceded by a distinct, much stronger, narrow peak site which lies more proximal to the TSS (**D** and **E**). This pattern is also observable in previously published articles mentioned above but has not been separately investigated so far. Therefore we flexibly identify the threshold that is best aligned to the expected relation to transcript levels and covers the more clearly distinguishable peak that lies more proximal to the TSS.

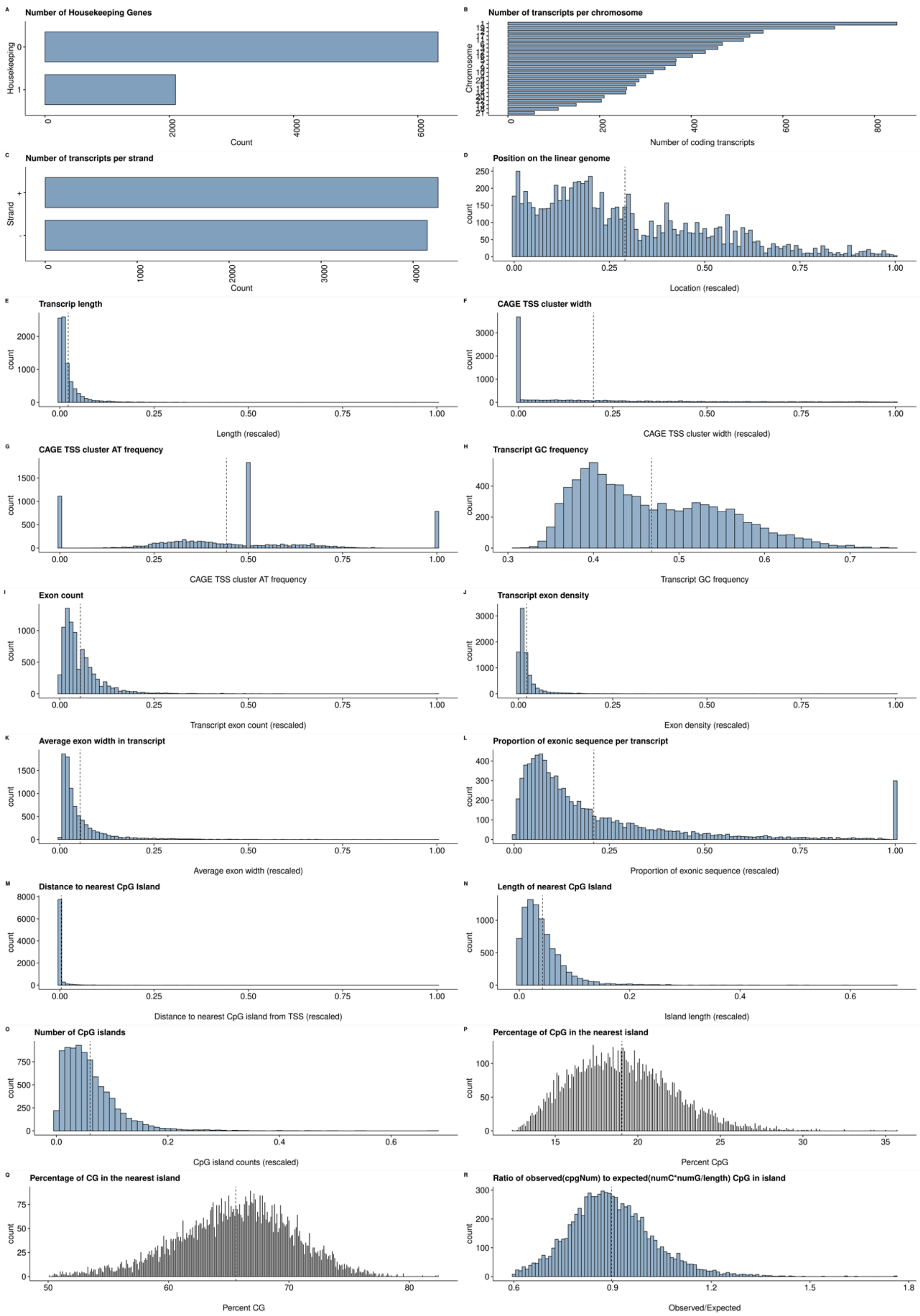

Supplementary Figure 3: Gene annotation and composition features (K562). Distribution of gene annotation and sequence composition features in the K562 cell line. Numeric features were rescaled to the range [0;1]. In sub-figures **A**-**C** the x-axes show the counts of features and the y-axes the feature values. In sub-figues **D**-**R** the x-axes show the feature values and the y-axes the counts of features.

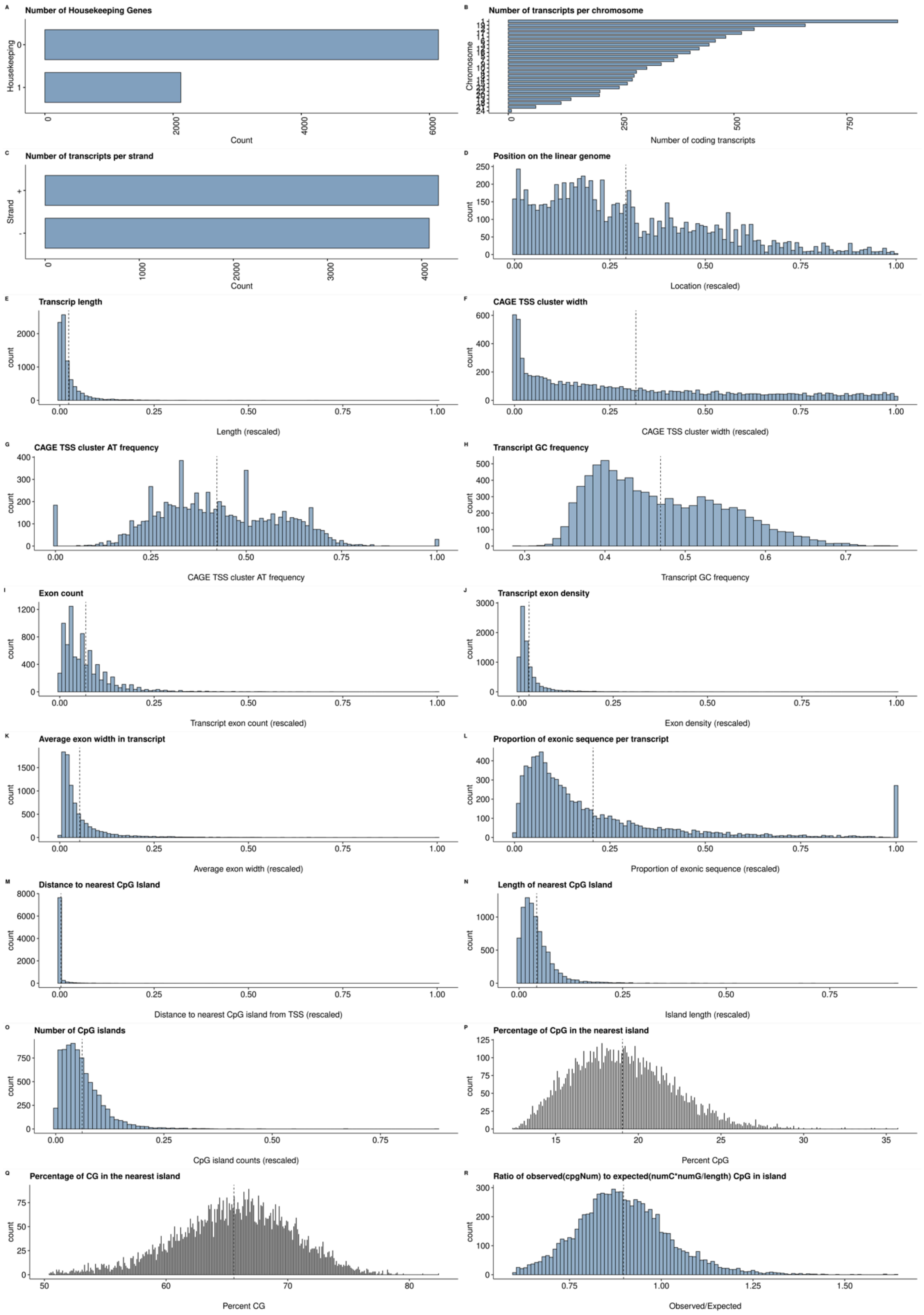

Supplementary Figure 4: Gene annotation and composition features (HepG2). See caption of supplementary figure 3 for more details.

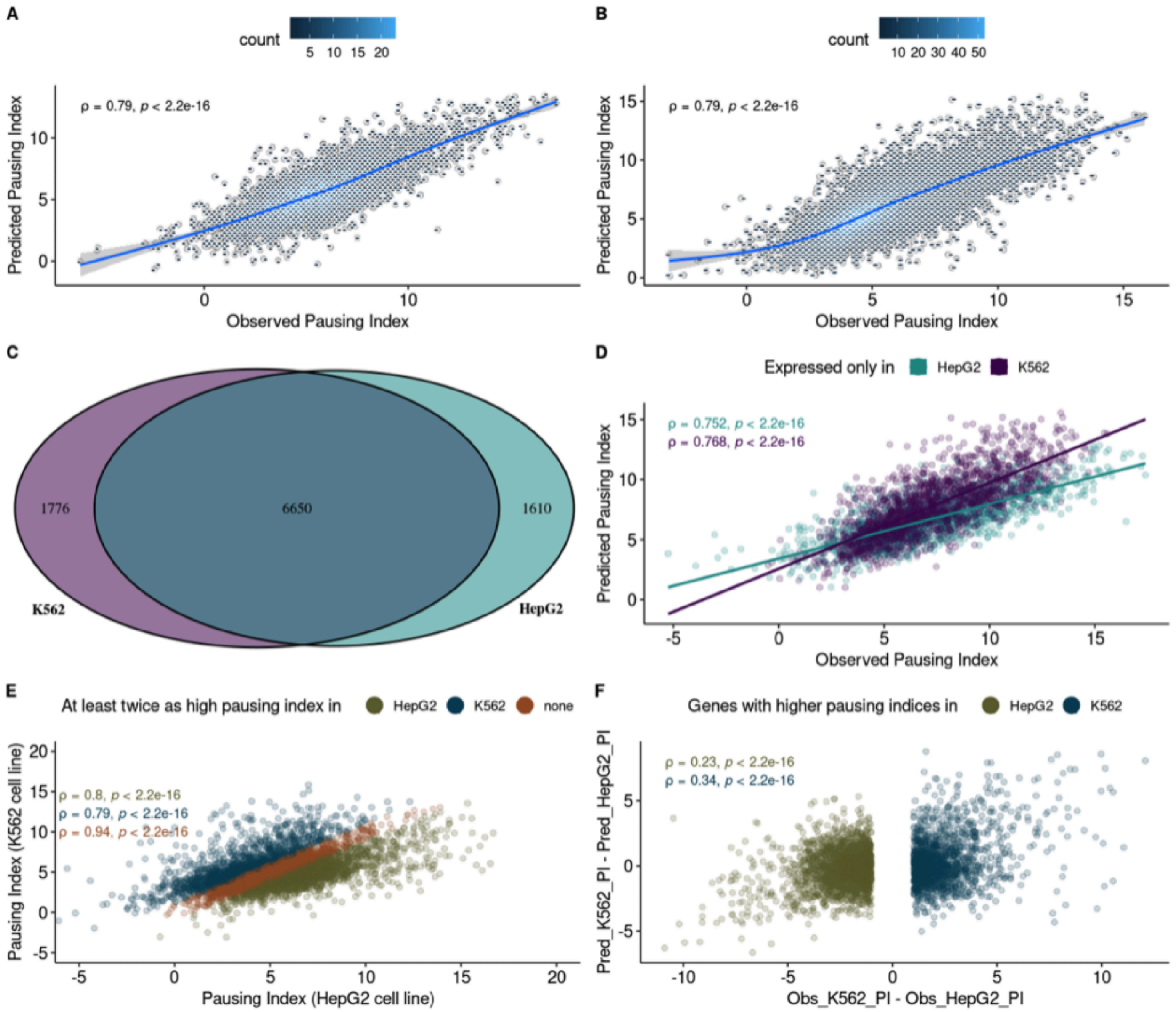

Supplementary Figure 5: Figure 2 analog for the HepG2 cell line. See caption of main figure 2 for more details.

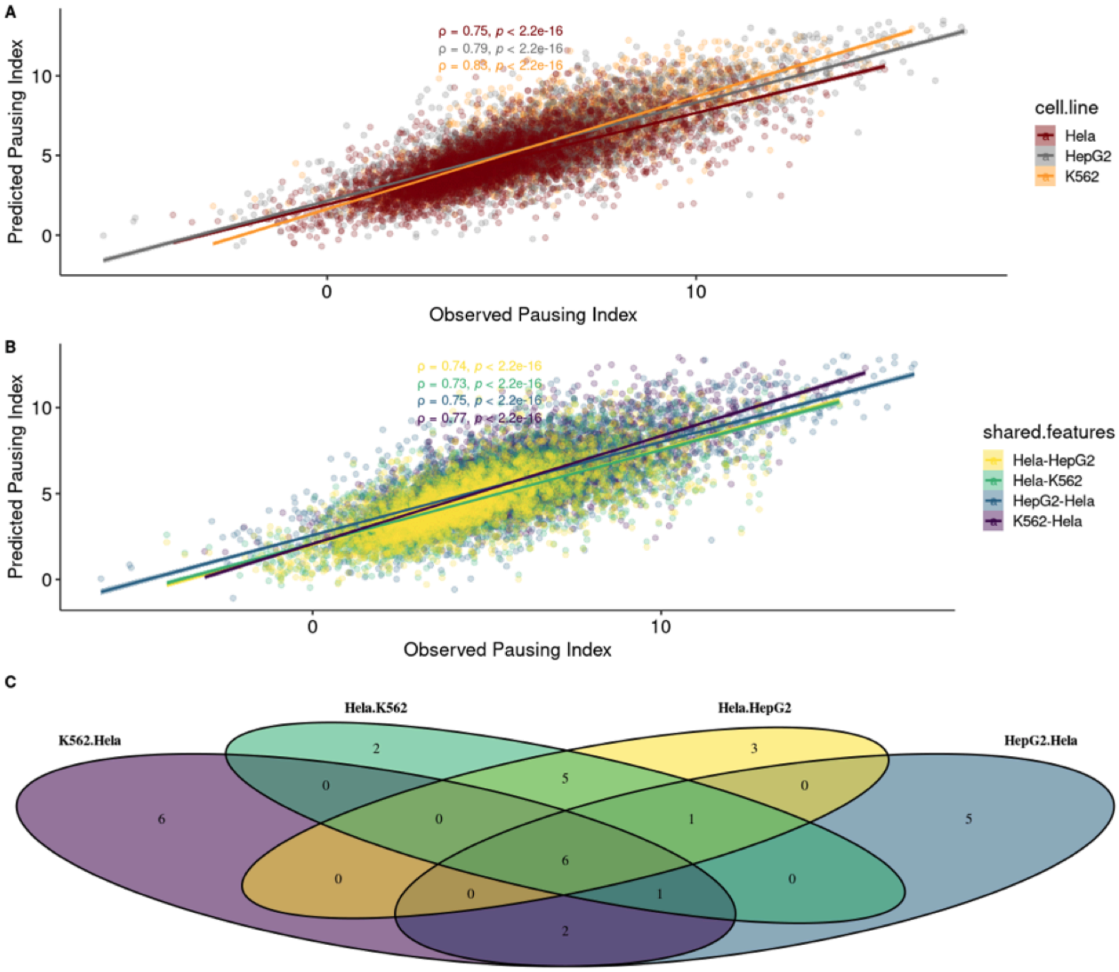

Supplementary Figure 6: **Model performance validation with the Hela cell line**. (**A**) Observed vs. predicted pausing indices (log2 scale) of 5-fold cross-validated and regularized XGB regression models in the K562 (yellow), HepG2 (gray) and Hela (red) cell line applied to an independent 50% hold-out test dataset from the same cell lines taken prior to training. Pearson's correlation coefficients rho (ρ) for each model with the associated p-values are depicted in the upper middle. The global Pearson's correlation coefficient rho (ρ) is depicted in the upper left. (**B**) Observed vs. predicted pausing indices (log2 scale) of models trained in analogy to (A) but with only features that are shared pairwise between cell lines. For instance, the "Hela-HepG2" model refers to a model trained and validated on the Hela data set based on features that are also available in the HepG2 cell line. (**C**) Venn diagram of top 15 ranking factors from each model shown in (**B**). The triple venn diagram of DNA-/RNA-binding factors from the K562, HepG2 and Hela cell lines. The Hela model trained on protein binding patterns of n=47 factors that are also present in the K562 data (Hela-K562) and validated on a 50% hold-out test data set taken prior to training, achieves an R-squared of 0.53 (see **Supplementary Figure S5 B**), a performance drop of only 0.03 percent points as compared to the full Hela model . Analogously, the Hela-HepG2 model (n=37 factors) achieves an R-squared of 0.54, the HepG2-Hela model (n=37 factors) an R-squared of 0.56 and the K562-Hela model (n=47 factors) an R-squared of 0.59, representing a performance drop in comparison to the full models of 0.02, 0.06 and 0.09, respectively.

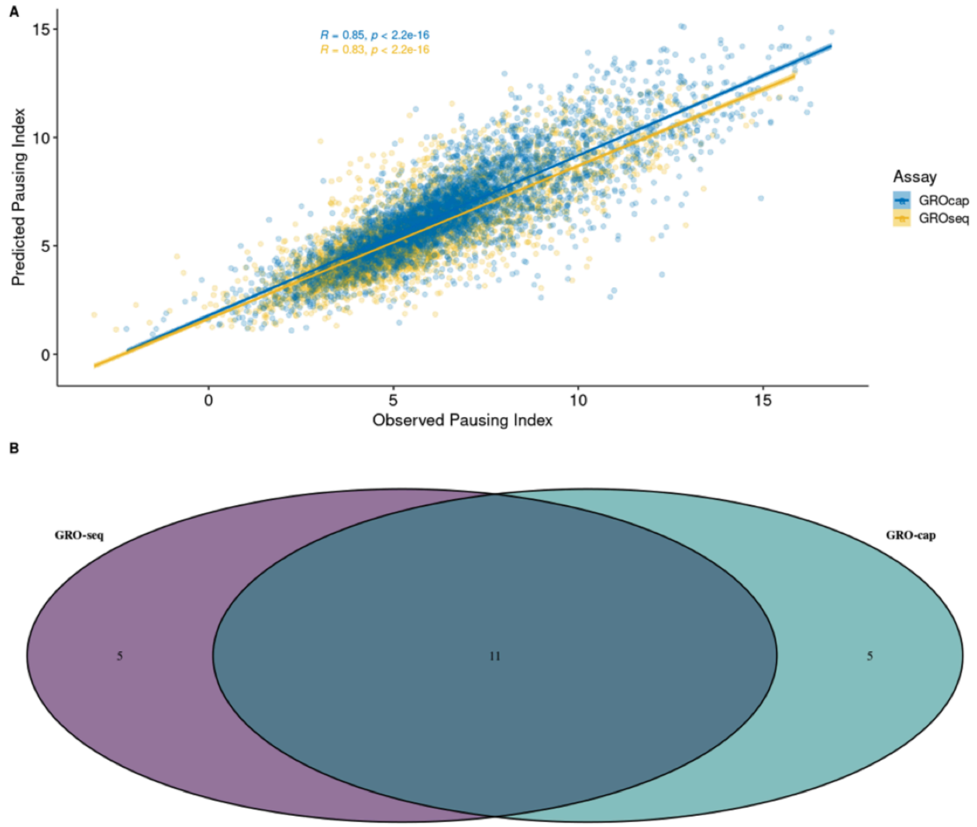

Supplementary Figure 7: Cross-technology evaluation with GRO-cap. (A) Observed vs. predicted pausing indices (log2 scale) of 5-fold cross-validated and regularized XGB regression models predicting the pausing index based on GRO-seq (yellow) and GRO-cap (blue) in the K562 cell line and applied to an independent 50% hold-out test dataset from the same cell line taken prior to training. Pearson's correlation coefficients rho (ρ) for each model with the associated p-values are depicted in the upper middle. The global Pearson's correlation coefficient rho (ρ) is depicted in the upper left. **(B)** Venn diagram of the top 16 ranking DNA-/RNA-binding factors from the K562 cell line across both sequencing protocols.

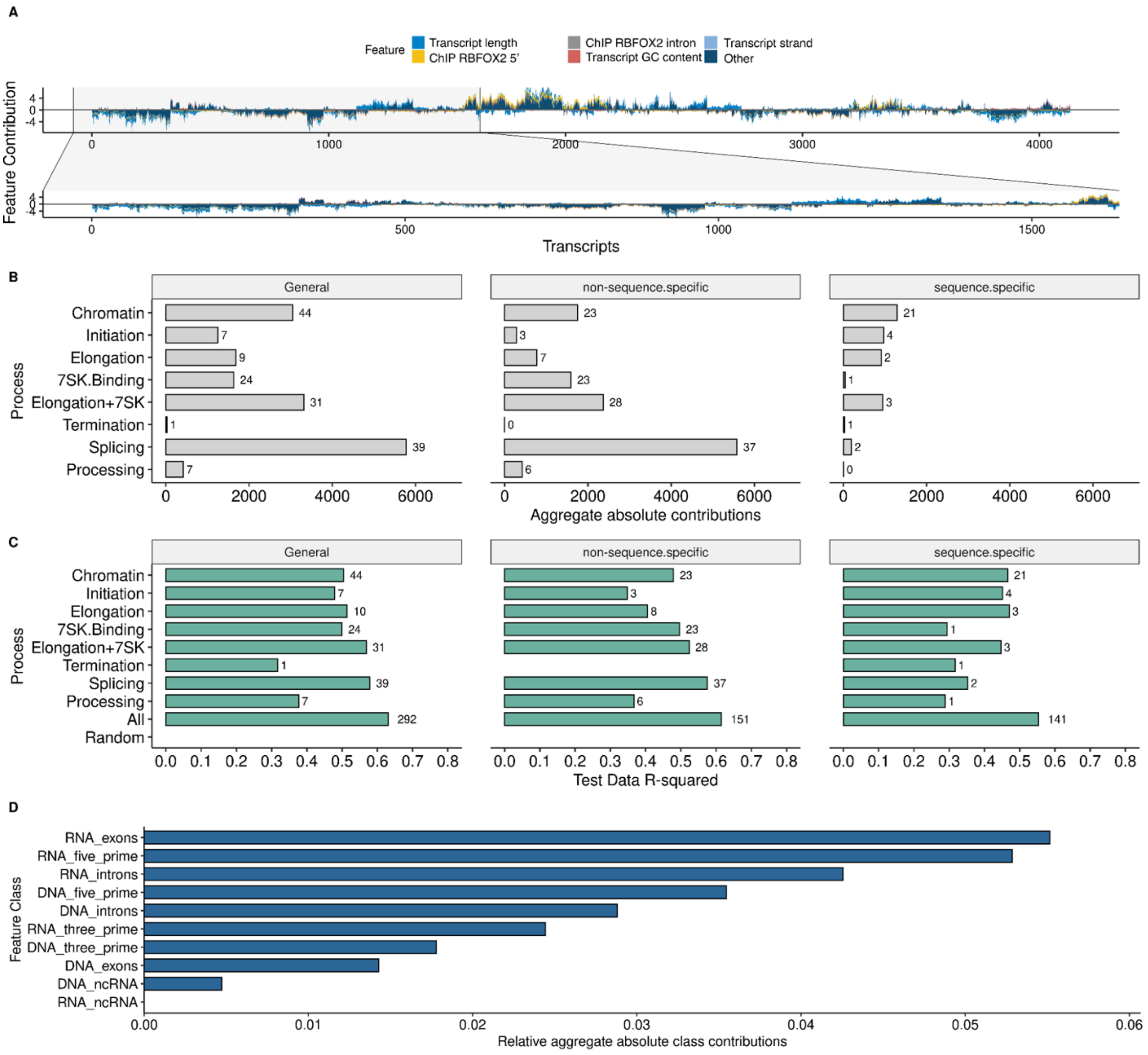

Supplementary Figure 8: Figure 3 analog for the HepG2 cell line. See caption of main figure 3 for more details.

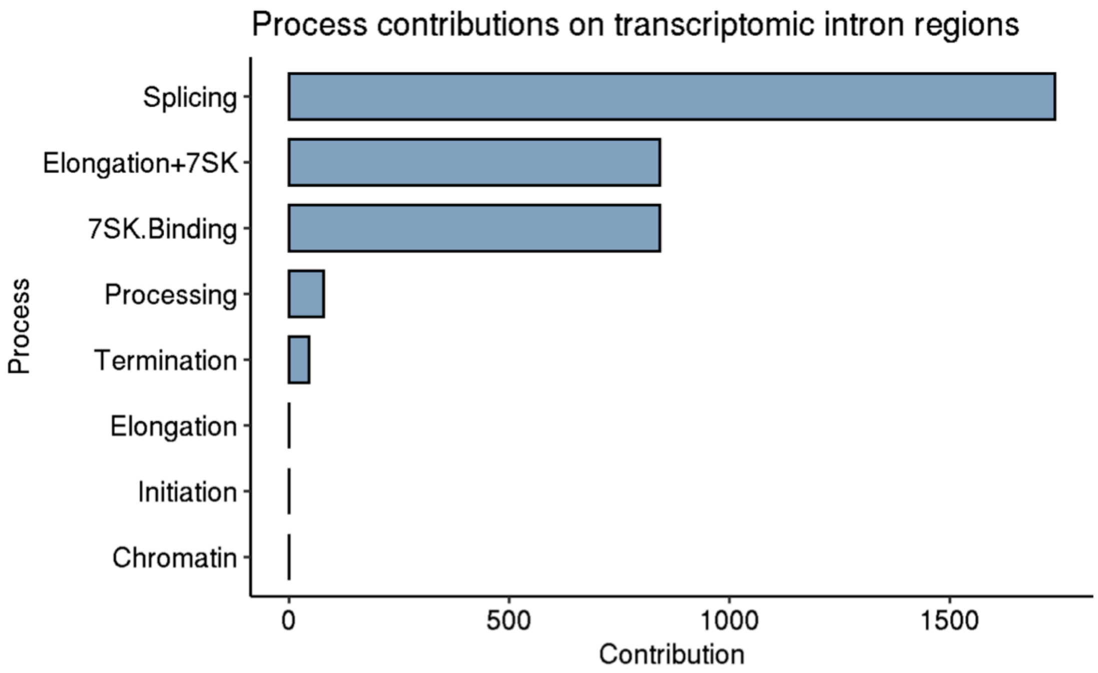

Supplementary Figure 9: Feature contributions on RNA introns (K562 cell line). Aggregate feature contributions (x-axis) of RNA intron binding factors by functional classes (y-axis).

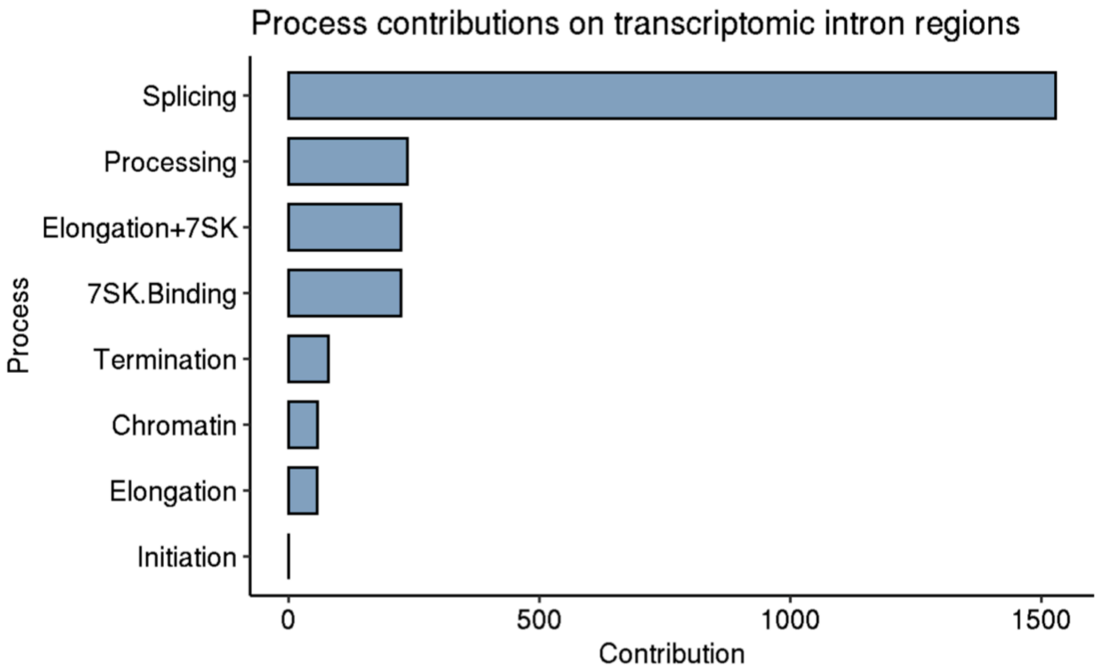

Supplementary Figure 10: Feature contributions on RNA introns (HepG2 cell line). See caption of supplementary figure 7 for more details.

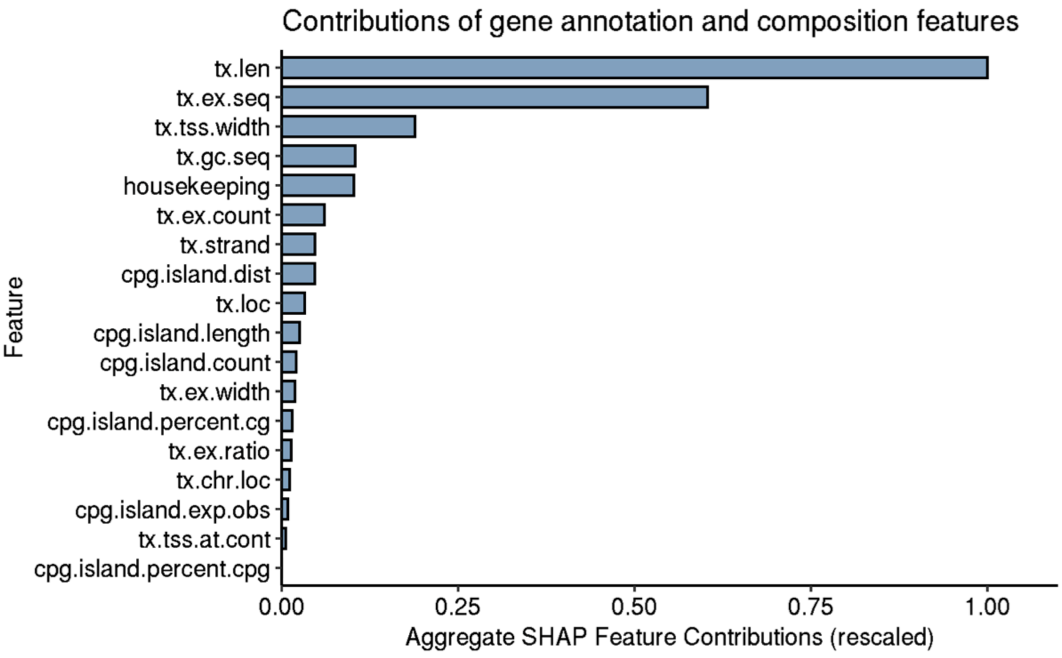

Supplementary Figure 11: Aggregate feature contributions of gene annotation and composition features (K562). Aggregate feature contributions (x-axis) of gene annotation and composition features (y-axis) in the K562 cell line.

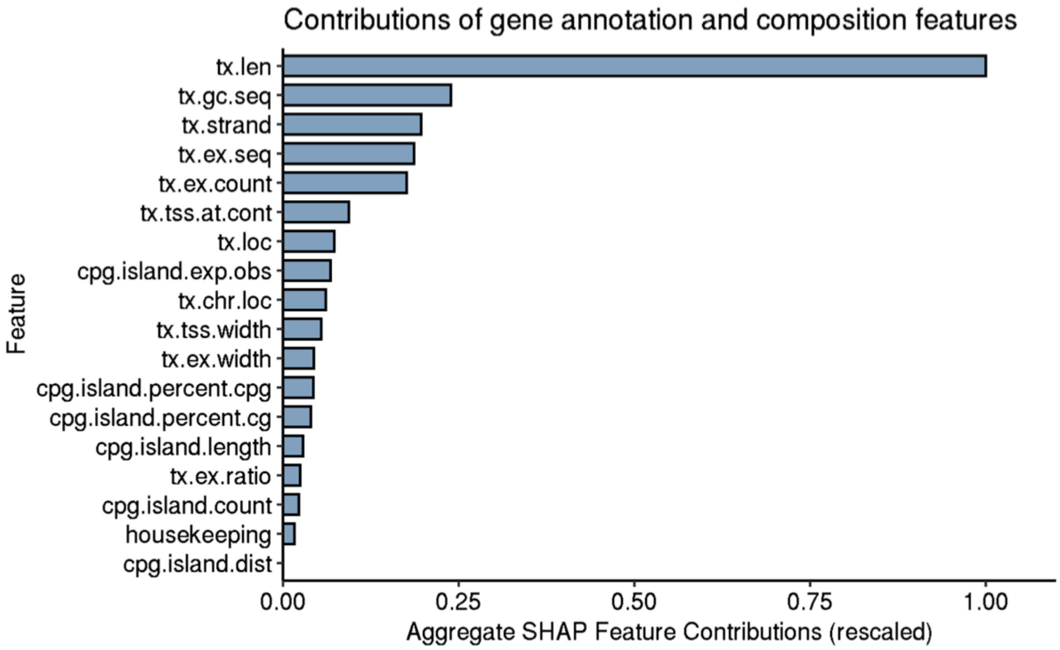

Supplementary Figure 12: Aggregate feature contributions of gene annotation and composition features (HepG2). See caption of supplementary figure 9 for more details.

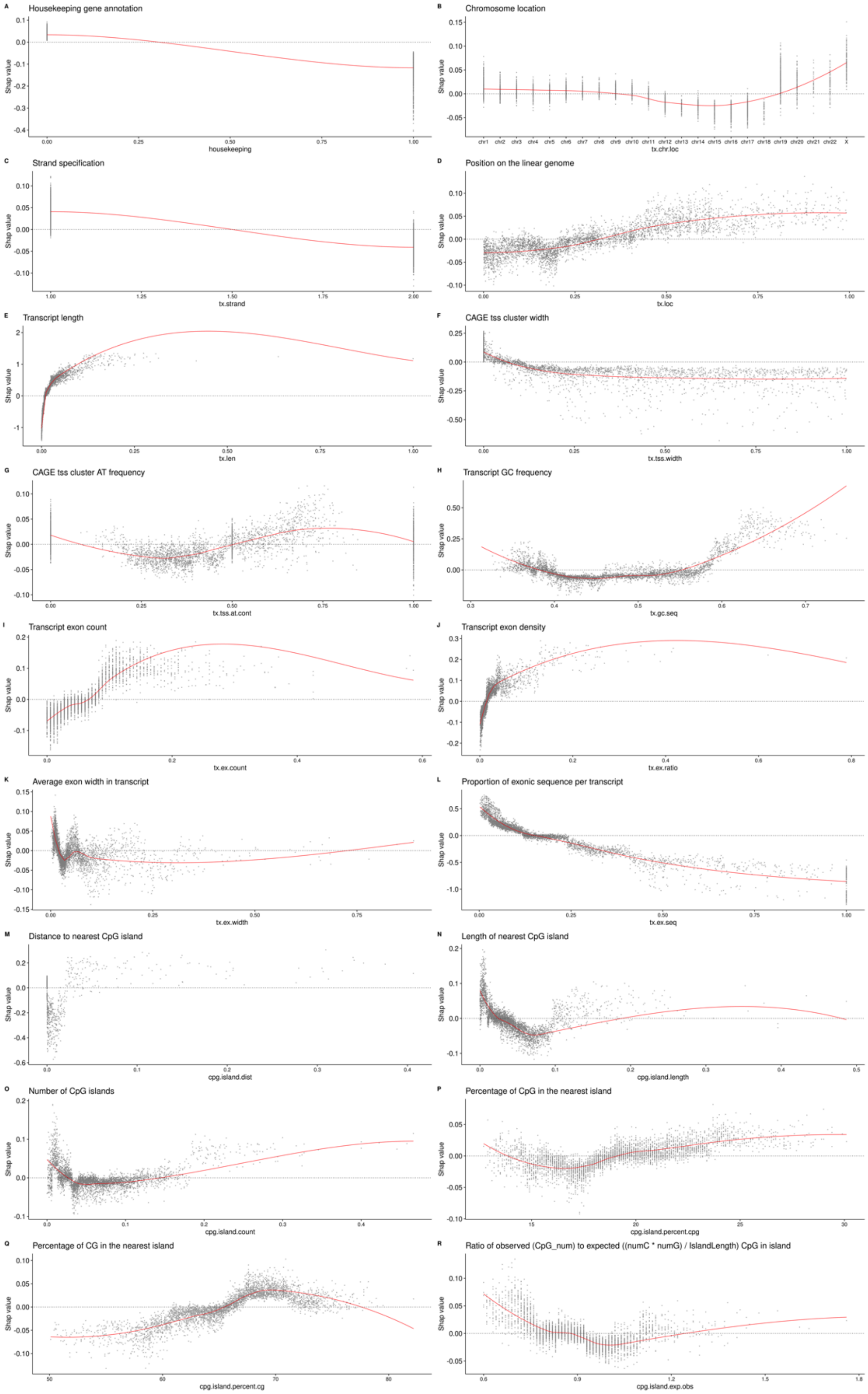

Supplementary Figure 13: Model feature contribution distributions of gene annotation and composition features (K562). Feature contributions (y-axes) of gene annotation and composition features values (x-axes) in the K562 cell line. For “tx.strand” feature, “1” denotes “+” (forward) strand and “2” denotes “-” (reverse strand).

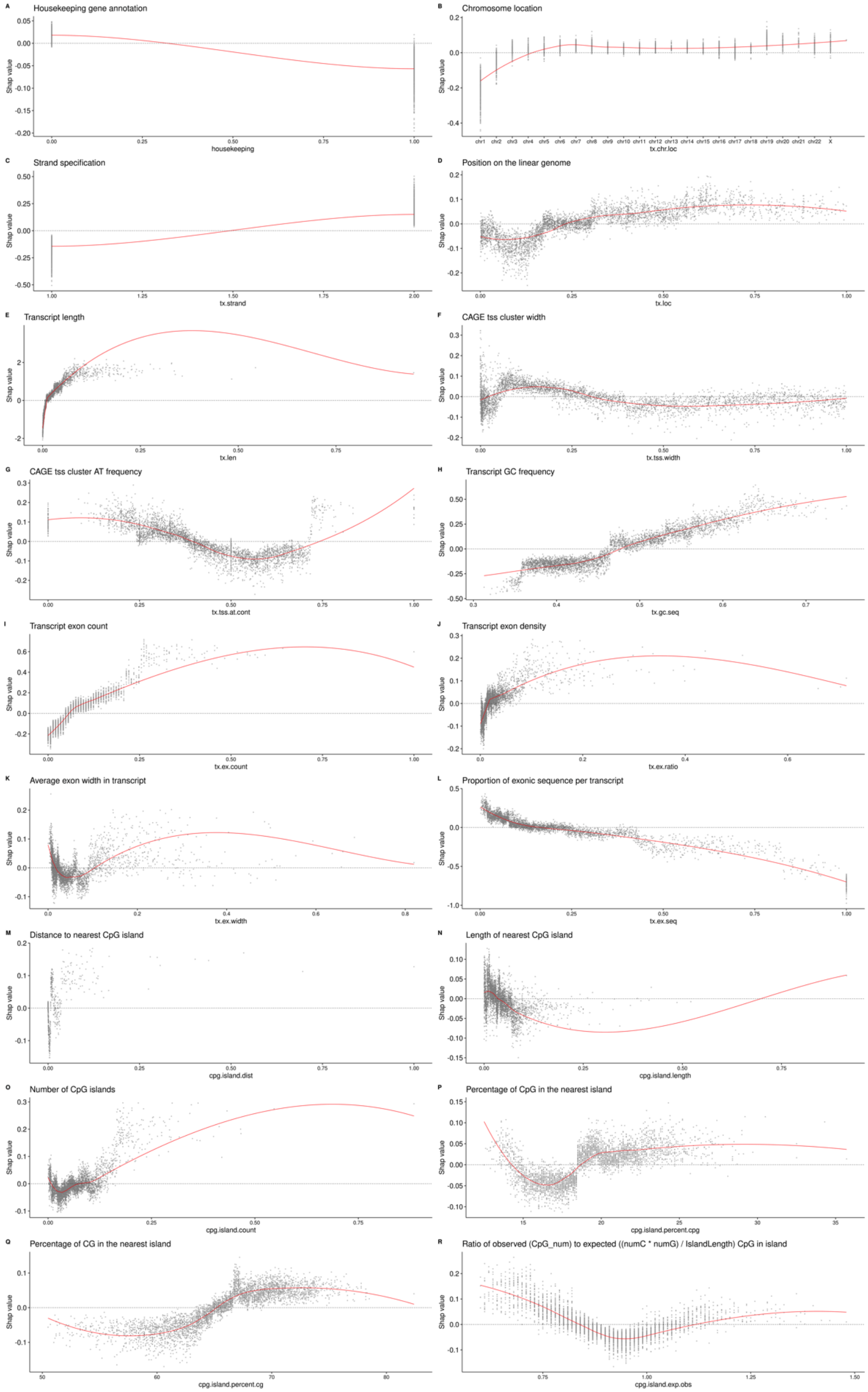

Supplementary Figure 14: Model feature contribution distributions of gene annotation and composition features (HepG2). See caption of supplementary figure 11 for more details.

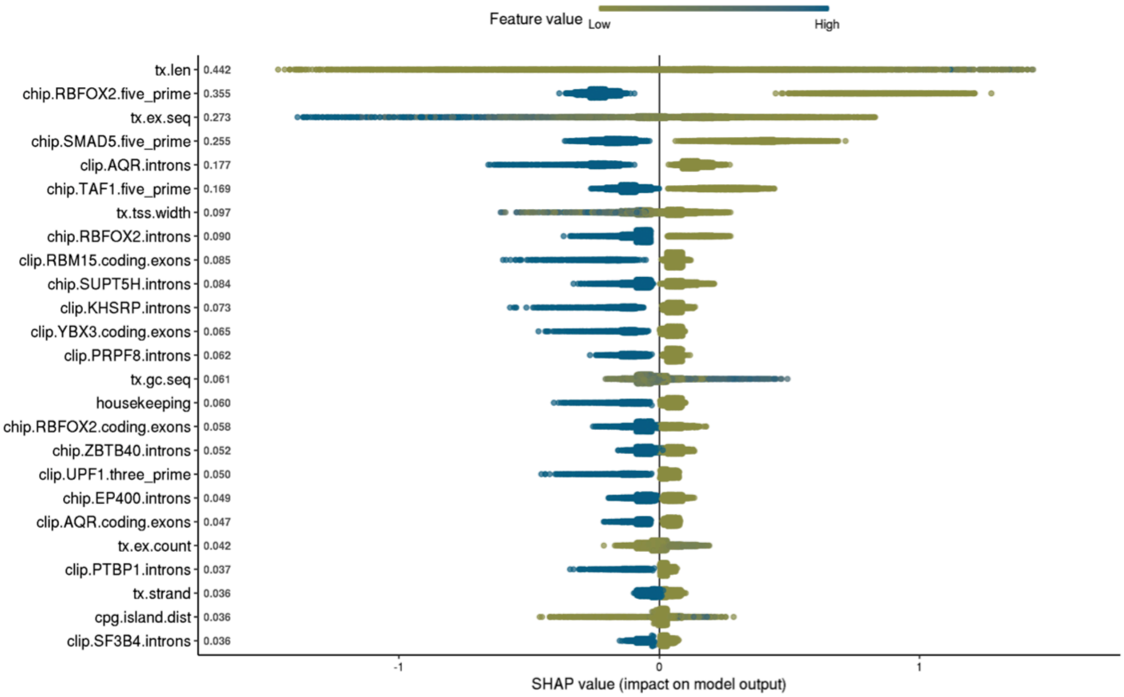

Supplementary Figure 15: Feature Contributions (K562). Feature contribution (x-axis) of the top 25 features (y-axis) from the full (*All)* individual K562 model.

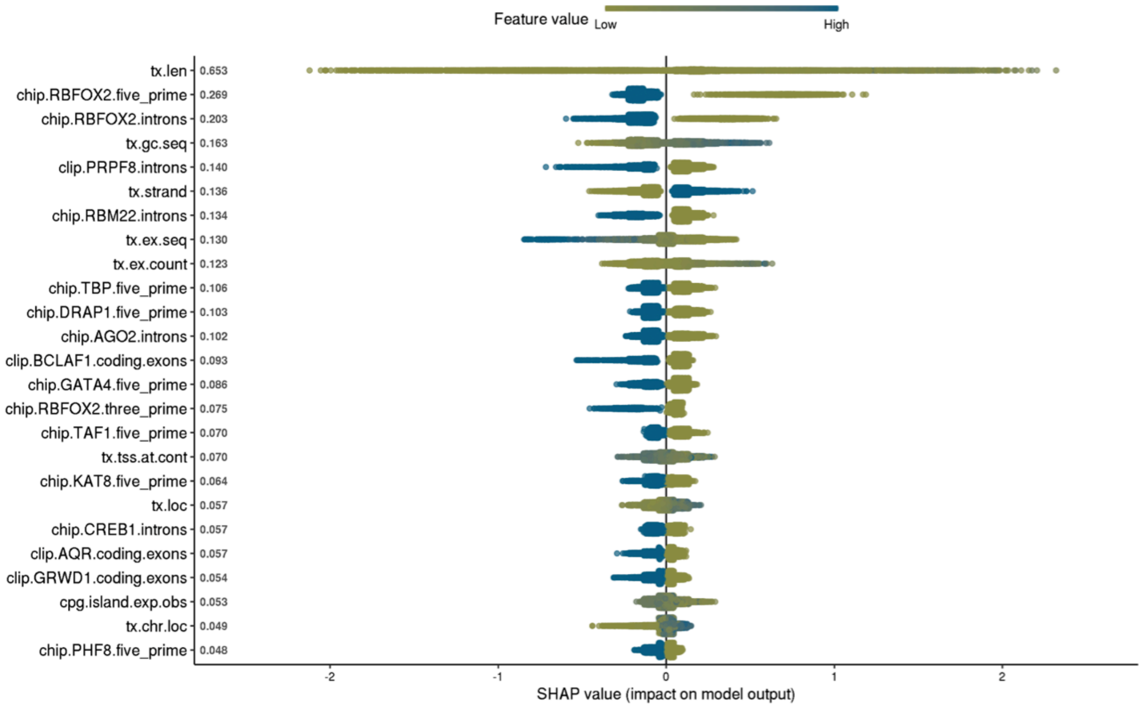

Supplementary Figure 16: Feature Contributions (HepG2). See caption of supplementary figure 13 for more details.

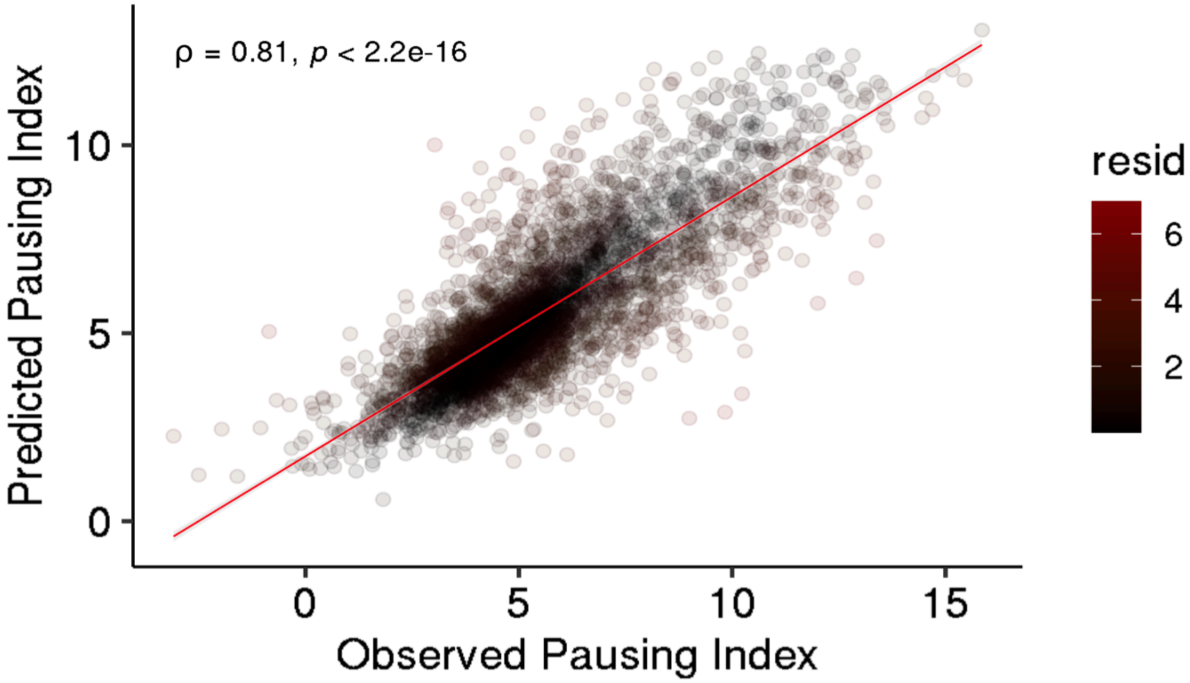

Supplementary Figure 17: Minimal model performance. Observed (x-axis) vs predicted (y-axis) pausing index of the 16 most influential factor model for the K562 cell line.

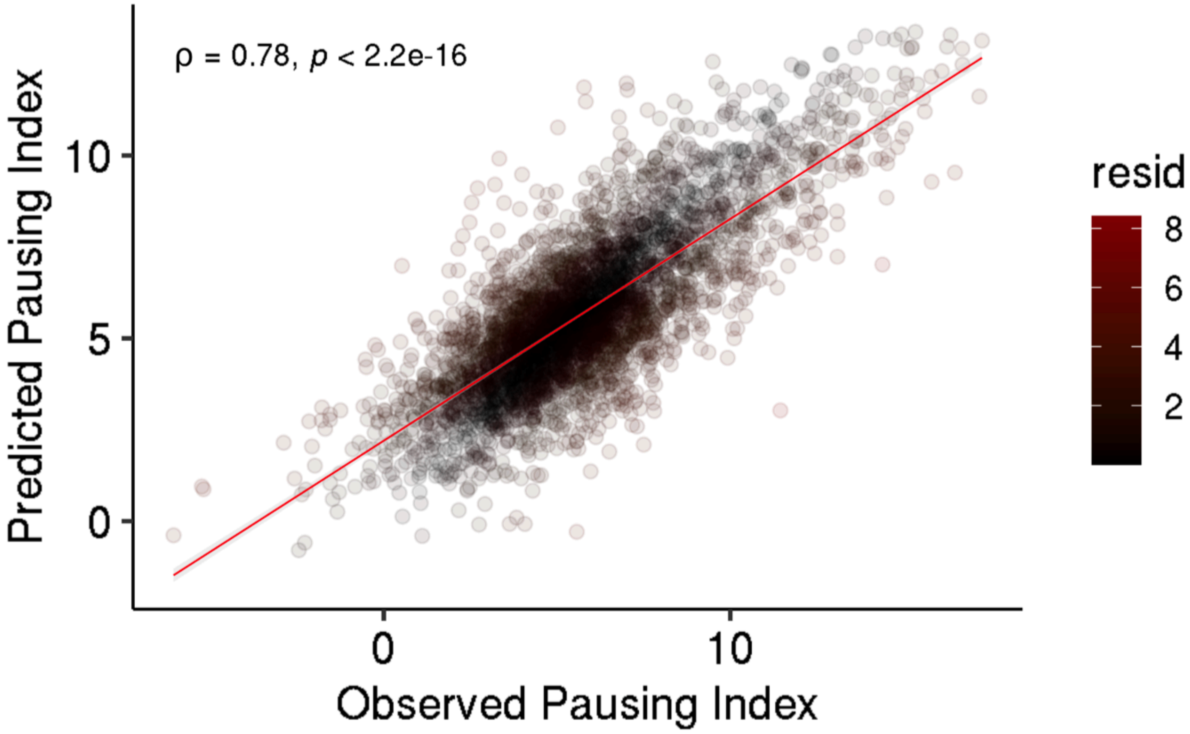

Supplementary Figure 18: Minimal model performance. Observed (x-axis) vs predicted (y-axis) pausing index of the 9 most influential factor model for the HepG2 cell line.

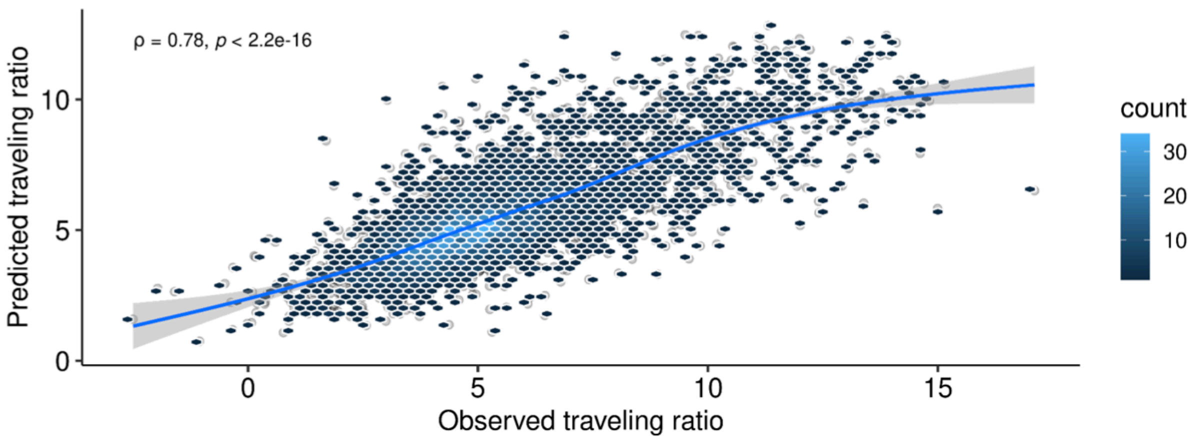

Supplementary Figure 19: Pol II only model. Observed vs. predicted pausing indices (log2 scale) of 5-fold cross-validated and regularized XGB regression models predicting the pausing index based on GRO-seq data in the K562 cell line and applied to an independent 50% hold-out test dataset from the same cell line taken prior to training with features for only the factors POLR2A, POLR2AphosphoS2, POLR2AphosphoS5, POLR2B, POLR2G and POLR2H. Pearson's correlation coefficients rho (ρ) with the associated p-values is depicted in the upper left.

SUPPLEMENTARY TABLES

Supplementary Table S1 (see xls file sheet “S1 K562 CHIPseq Factors”) : List of factors from the ENCODE CHIP-seq experiments for the K562 cell line.

Supplementary Table S2 (see xls file sheet “S2 HepG2 CHIPseq Factors”) : List of factors from the ENCODE CHIP-seq experiments for the HepG2 cell line.

Supplementary Table S3 (see xls file sheet “S3 Hela CHIPseq Factors”) : List of factors from the ENCODE CHIP-seq experiments for the Hela cell line.

Supplementary Table S4 (see xls file sheet “S4 K562 CHIPseq Accessions”) : List of ENCODE CHIP-seq experiment accession numbers for the K562 cell line.

Supplementary Table S5 (see xls file sheet “S5 HepG2 CHIPseq Accessions”): List of ENCODE CHIP-seq experiment accession numbers for the HepG2 cell line.

Supplementary Table S6 (see xls file sheet “S6 Hela CHIPseq Accessions”): List of ENCODE CHIP-seq experiment accession numbers for the Hela cell line.

Supplementary Table S7 (see xls file sheet “S7 K562 eCLIPseq Factors”): List of factors from the ENCODE eCLIP-seq experiments for the K562 cell line.

Supplementary Table S8 (see xls file sheet “S8 HepG2 eCLIPseq Factors”): List of factors from the ENCODE eCLIP-seq experiments for the HepG2 cell line.

Supplementary Table S9 (see xls file sheet “S9 Hela POSTAR RNA-binding Factors”): List of factors from the POSTAR CLIP data base for the Hela cell line.

Supplementary Table S10 (see xls file sheet “S10 K562 eCLIPseq Accessions”) : List of ENCODE eCLIP-seq experiment accession numbers for the K562 cell line.

Supplementary Table S11 (see xls file sheet “S11 HepG2 eCLIPseq Accessions”): List of ENCODE eCLIP-seq experiment accession numbers for the HepG2 cell line.

Supplementary Table S12 (see xls file sheet “S12 K562 7SK Binding Factors”): List of factors that bind the 7SK ncRNA in the K562 cell line. Please note that binding signals of pseudo 7SK ncRNA transcript variants expressed above median ncRNA expression levels were included. Their consideration was supported by the transcripts' high mean pairwise sequence similarity (41) of 0.74 and high mean conservation score of 923.58 (PAM250 scoring matrix) resulting from a multiple sequence alignment (ClustalW alignment) of corresponding 7SK transcripts.

Supplementary Table S13 (see xls file sheet “S13 HepG2 7SK Binding Factors”): List of factors that bind the 7SK ncRNA in the HepG2 cell line. Please note that binding signals of pseudo 7SK ncRNA transcript variants expressed above median ncRNA expression levels were included. Their consideration was supported by the transcripts' high mean pairwise sequence similarity (41) of 0.81 and high mean conservation score of 302.29 (PAM250 scoring matrix) resulting from a multiple sequence alignment (ClustalW alignment) of corresponding 7SK transcripts.

Supplementary Table S14 (see xls file sheet “S14 Hela 7SK Binding Factors”): List of factors that bind the 7SK ncRNA in the Hela cell line. Please note that binding signals of pseudo 7SK ncRNA transcript variants expressed above median ncRNA expression levels were included. Their consideration was supported by the transcripts' high mean pairwise sequence similarity (41) of 0.69 and high mean conservation score of 1919.71 (PAM250 scoring matrix) resulting from a multiple sequence alignment (ClustalW alignment) of corresponding 7SK transcripts.

Supplementary Table S15 (see xls file sheet “S15 K562 Factor Bindings”): Number of bindings on genomic and transcriptomic transcript regions per factor in the K562 cell line.

Supplementary Table S16 (see xls file sheet “S16 HepG2 Factor Bindings”): Number of bindings on genomic and transcriptomic transcript regions per factor in the HepG2 cell line.

Supplementary Table S17 (see xls file sheet “S17 Hela Factor Bindings”): Number of bindings on genomic and transcriptomic transcript regions per factor in the Hela cell line.

Supplementary Table S18 (see xls file sheet “S18 Known Pausing Factors”): List of known pausing factors from the literature.

Supplementary Table S19 (see xls file sheet "S19 K562 Factors per Process"): List of factors in the K562 cell line per functional process.

Supplementary Table S20 (see xls file sheet "S20 HepG2 Factors per Process"): List of factors in the HepG2 cell line per functional process.

Supplementary Table S21 (see xls file sheet “S21 K562 Sequence Specificity”): An indicator matrix whether a factor in the K562 cell line is sequence specific (column *SS*), non-sequence specific (column *NSS*), a RNA-binding factor (column *RBP*) or a DNA-binding factor (column DBP).

Supplementary Table S22 (see xls file sheet “S22 HepG2 Sequence Specificity”): An indicator matrix whether a factor in the HepG2 cell line is sequence specific (column *SS*), non-sequence specific (column *NSS*), a RNA-binding factor (column *RBP*) or a DNA-binding factor (column DBP).

Supplementary Table S23 (see xls file sheet "S23 Subspace Factors Presence"): An indicator matrix whether a factor was present in any of the feature subspaces. “1” denotes present, “0” denotes” not present

Supplementary Table S24 (see xls file sheet “S24 Hyperparameters”): Specification of hyperparameters of the Extreme Gradient Boosting Tree regressor.

Supplementary Table S25 (see xls file sheet “S25 All Model Results”): Model results for each cell line and each feature subspace. Column *subspace* gives the feature subspace the model was trained on. An appendix of “*ss*” to the feature subspace name indicates a model trained on binding features of sequence specific factors and “*nss”* of non-sequence specific factors. The model type *synchronised.model.matrices* as opposed to *individual.model.matrices* refers to a model that was trained on features observed in both of the cell lines (K562 and HepG2). Column *train.rsqrd* gives the R^2 performance of the 5-fold cross-validation procedure. Column *test.rsqrd* gives the performance on a 50% hold out test data set taken before training. Column *mean.shap* gives the average feature contribution over all factor associated binding features.

Supplementary Table S26 (see xls file sheet “S26 Model Type Performances”):

Model type performance comparison table, giving the R-squared values for a model trained in a cell line given in column “Train” with the training/testing approach given in “Strategy” and the test data set given in column “Test for each model type in the remaining columns afterwards (Ridge Regression, Random Forest, Gradient Boosting Decision Trees and Extreme Gradient Boosting Decision Trees).

Supplementary Table S27 (see xls file sheet “S27 Data Accessions”): List of data accession numbers.
